# Chaperonin‐containing TCP1 subunit 6A inhibition via TRIM21‐mediated K48‐linked ubiquitination suppresses triple‐negative breast cancer progression through the AKT signalling pathway

**DOI:** 10.1002/ctm2.70097

**Published:** 2024-11-18

**Authors:** Mengdi Yang, Jianing Cao, Tiantian Liu, Bin Li, Jinyan Wang, Shuangyue Pan, Duancheng Guo, Zhonghua Tao, Xichun Hu

**Affiliations:** ^1^ Department of Breast and Urologic Medical Oncology Fudan University Shanghai Cancer Center Shanghai P. R. China; ^2^ Department of Oncology Shanghai Medical College Fudan University Shanghai P. R. China

**Keywords:** CCT6A, immunotherapy, TRIM21, triple‐negative breast cancer

## Abstract

**Background:**

Triple‐negative breast cancer (TNBC) is distinguished by a significant likelihood of distant recurrence and an unfavourable prognosis. However, the underlying molecules and mechanisms have not been fully elucidated.

**Methods:**

We investigated the expression profile and clinical relevance of chaperonin‐containing TCP1 subunit 6A (CCT6A) in TNBC. We performed cell function assays on TNBC cells with CCT6A knockdown or overexpression. To further explore the mechanism of action of CCT6A, RNA sequencing and co‐immunoprecipitation–mass spectrometry analyses were utilized. Rescue and ubiquitination assays evaluated the impact of TRIM21‐mediated CCT6A ubiquitination and degradation on TNBC progression in vitro and in vivo. Finally, we studied the potential of Ipatasertib, a pharmacological AKT inhibitor, and/or anti‐PD1 therapy in inhibiting TNBC progression.

**Results:**

Elevated CCT6A expression in TNBC patients was associated with an adverse prognosis and lymph node metastasis. Mechanistically, CCT6A facilitated cell migration, invasion, epithelial‐mesenchymal transition and proliferation by activating the phosphatidylinositol 3‐kinase (PI3K)/AKT pathway. The TRIM21 RING domain is an E3 ligase, facilitating the K48‐linked ubiquitination‐mediated degradation of CCT6A, thereby impeding TNBC progression. Moreover, in the tumour tissues of the CCT6A‐overexpressing mice, the quantity of CD8+ T cells and the concentration of secreted interferon‐gamma were decreased, whereas in the group double‐overexpression of CCT6A and TRIM21, they were elevated; the opposite was observed in the knockdown and double‐knockdown groups. Ipatasertib demonstrated enhanced efficacy in inhibiting cell proliferation, invasion and migration in TNBC cells ectopically expressing CCT6A. When Ipatasertib and anti‐PD1 therapies were combined, both the tumour volume and mass exhibited a notable reduction, while the expression of CD45+CD8+ T cells increased, and that of CD45+CD4+CTLA4+ and CD45+CD4+PD1+ T cells decreased.

**Conclusions:**

Our findings indicate that TRIM21 inhibits TNBC progression by facilitating the K48‐linked ubiquitination‐mediated degradation of CCT6A via the PI3K/AKT signalling pathway. This highlights the potential of Ipatasertib and/or anti‐PD1 as therapeutic strategies, particularly for TNBC patients overexpressing CCT6A.

**Key points:**

- Chaperonin TCP1 subunit 6A (CCT6A) plays an oncogenic role in triple‐negative breast cancer (TNBC) through the AKT signaling pathway.
- TRIM21 facilitated K48‐linked ubiquitination‐mediated degradation of CCT6A, thereby impeding TNBC progression.
- Our study collectively underscores the potential of Ipatasertib in conjunction with anti‐PD1 therapy as a promising strategy to counteract CCT6A/AKT hyperactivity‐driven TNBC progression.

## Graphical abstract

1. Chaperonin TCP1 subunit 6A (CCT6A) plays an oncogenic role in triple‐negative breast cancer (TNBC) through the AKT signaling pathway.
2. TRIM21 facilitated K48‐linked ubiquitination‐mediated degradation of CCT6A, thereby impeding TNBC progression.
3. Our study collectively underscores the potential of Ipatasertib in conjunction with anti‐PD1 therapy as a promising strategy to counteract CCT6A/AKT hyperactivity‐driven TNBC progression.

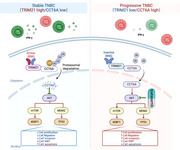

## 1 INTRODUCTION

Breast cancer ranks among the prevalent forms of cancer globally, and its incidence is increasing worldwide.^1^ Triple‐negative breast cancers (TNBCs) represent 15%–20% of total breast cancer cases and are associated with early metastatic recurrence and worse patient outcomes.^2^ Due to its diverse genetic makeup and absence of identifiable molecular targets, only slight advancements have been achieved in enhancing survival rates for TNBC individuals.^3^ While the key molecules and mechanisms underlying the aggressive phenotype of TNBC have been partially elucidated, further investigations are still needed to fully clarify this complex process.^4^ Therefore, potential targets for TNBC diagnosis and treatment should be identified.^2^

Chaperonin‐containing TCP1 subunit 6A (CCT6A) serves as a constituent of the chaperonin‐containing TCP1 (CCT) complex. Earlier research indicates that around 5%–10% of recently produced cytoplasmic proteins transit via the CCT chaperone, underscoring its critical function in organizing the cytoskeleton.^5^, ^6^ CCT6A has been acknowledged as a unique element in the innate ERK 1/2 signalling complex.^7^ Research has established the tumour‐promoting role of CCT6A in a variety of tumours. CCT6A was found to inhibit transforming growth factor beta (TGF‐β)‐mediated metastatic signalling in lung cancer by inhibiting SMAD2.^8^ Moreover, CCT6A was linked to unfavourable prognosis and tumour metastasis^9^ and with reduced immune infiltration in colorectal cancer.^10^ In the progression of breast cancer and hepatocellular carcinoma, CCT6A plays a vital role.^11^ However, the mechanisms of CCT6A in BC, especially TNBC, need further exploration. The phosphatidylinositol 3‐kinase (PI3K)/AKT pathway contributes to tumour occurrence and progression.^12^ Zeng et al. reported that CCT6A knockdown suppressed osteosarcoma cell growth and AKT pathway activation in vitro.^13^ However, relatively few related studies have been conducted, and further in‐depth research is needed.

The TRIM family proteins feature a preserved N‐terminus housing a unique RING motif, one or two zinc‐finger regions called B‐boxes and a coiled‐coil domain.^14^ The TRIM21 gene, positioned on chromosome 11, encodes a widely distributed protein recognized as a RING‐dependent E3 ligase present in both the cytoplasm and nucleus.^15^ Recent investigations have revealed diminished TRIM21 expression in breast cancer.^16^ TRIM21 ubiquitinates talin1 to regulate breast cancer cell adhesion and tumour metastasis.^17^ TRIM21‐mediated proteolytic regulation of CD73 orchestrates tumour immunogenicity.^18^ However, the role and mechanism of the TRIM21/CCT6A/AKT axis in TNBC are still unknown.

Our findings revealed that CCT6A expression is elevated in TNBC, which is correlated with an unfavourable prognosis among patients. Functionally, CCT6A stimulates epithelial‐mesenchymal transition (EMT), cell migration, invasion and cell proliferation, while concurrently suppressing apoptosis by activating the AKT signalling pathway. Notably, TRIM21, an interacting partner of CCT6A, is downregulated in TNBC, orchestrating the K48‐linked ubiquitination of CCT6A and its subsequent degradation via the ubiquitin‐proteasome pathway. This mechanism effectively impedes TNBC progression while increasing the CD8+ T cell count and interferon‐gamma (IFN‐γ) secretion. Moreover, TRIM21 antagonizes the oncogenic actions of CCT6A both in vivo and in vitro. Notably, in our study, the combination of Ipatasertib and anti‐PD1 therapy significantly increased the population of CD45+CD8+ T cells, which was concurrently accompanied by decreased levels of the CD45+CD4+CTLA4+ and CD45+CD4+PD1+ T cell subpopulations. These findings collectively underscore the potential of Ipatasertib in conjunction with anti‐PD1 therapy as a promising strategy to counteract CCT6A/AKT hyperactivity‐driven TNBC progression. Our study identifies CCT6A as a target of ubiquitination by TRIM21. This breakthrough reveals promising directions for manipulating protein modifications in breast cancer treatment. Furthermore, we highlight our groundbreaking revelation of the involvement of CCT6A in p53 regulation, offering innovative insights for p53‐associated investigations.

## 2 MATERIALS AND METHODS

### 2.1 Patients

One hundred eight TNBC patients who underwent surgery between August 2015 and December 2017 without previous systemic neoadjuvant treatment at Fudan University Shanghai Cancer Center (FUSCC) were obtained. Patients aged between 18 and 70 years, with histologically confirmed TNBC from either the primary or metastatic tumour, and without prior systemic treatment for metastatic disease were considered eligible. Meeting an Eastern Cooperative Oncology Group Performance Status of 0 or 1 was another essential eligibility criterion. TNBC patient categorization adhered to the NCCN^8th^ guidelines, with diagnoses confirmed histologically by a specialized pathologist for all individuals. Every individual received a histological diagnosis from a skilled pathologist. All patients provided written informed consent, and the research procedures received approval from the FUSCC Ethics Review Board.

### 2.2 Cell culture and lentivirus packaging

Obtained from the Chinese Academy of Sciences cell repository, TNBC cell lines 4T1, BT‐549 and MDA‐MB‐231 were utilized. MDA‐MB‐231 cells were maintained at 37°C in a humidified atmosphere with 5% CO_2_ using Dulbecco's Modified Eagle Medium with 10% fetal bovine serum (FBS) added. 4T1 cells and BT‐549 were cultured in RPMI‐1640 medium with the same supplements as described above. The protocol for lentivirus packaging was described previously.^19^ 4T1*^shCct6a^* and MDA‐MB‐231^shCCT6A^ cells were repacked lentivirus with shTRIM21, thereby establishing double‐knockout stable transfectants 4T1*^shCct6a+shTrim21^* and MDA‐MB‐231^shCCT6A+shTRIM21^. 4T1*^Cct6a^* and BT‐549^CCT6A^ was repackaging lentivirus of TRIM21 or TRIM21‐ΔRING, thereby establishing double‐overexpression stable transfectants 4T1*^Cct6a+Trim21^*, 4T1*^Cct6a+Trim21‐ΔRING^*, BT‐549^CCT6A+TRIM21^ and BT‐549^CCT6A+TRIM21‐ΔRING^ cell lines. Then we confirmed the expression of CCT6A and TRIM21 in indicated stable cells by western blotting. The plasmid sequences can be found in Table S1. A Mycoplasma Kit from Sigma‒Aldrich was used to detect mycoplasma presence.

### 2.3 Western blotting analysis and co‐immunoprecipitation

Following rinsing with 1× phosphate‐buffered saline (PBS), the cells were lysed in radioimmunoprecipitation assay lysis buffer containing protease inhibitors. Subsequently, the protein concentrations in the lysates were gauged with an improved BCA kit (#P0012, Beyotime). The proteins within the cell lysates were then segregated through sodium dodecyl sulfate‐polyacrylamide gel electrophoresis, transferred to onto polyvinylidene fluoride membranes and tested with antibodies for TRIM21 (#92043, CST); CCT6A (#ab110905, Abcam); the EMT markers Slug (#9585, CST), Vimentin (#5741, CST), E‐cadherin (#3195, CST), ZEB1 (#21544‐1‐AP, Proteintech); p‐P53 (#9284, CST), mTOR (#ET1608‐5, HUABIO), BAX (#ET1603‐34, HUABIO), p‐mTOR (#HA60094, HUABIO), P53 (#2527, CST), PI3K (#4249, CST), BCL2 (#ET1702‐53, HUABIO), MDM2 (#82504, CST), AKT (#ET1609‐51, HUABIO), p‐AKT (#4060, CST) and glyceraldehyde 3‐phosphate dehydrogenase (GAPDH) (#60004‐1‐AP, Proteintech). In immunoprecipitation (IP) experiments, protein lysates were mixed with antibodies or with control immunoglobulin G and incubated overnight at 4°C with stirring. The following day, this solution was added to protein A/G beads for 2–3 h at room temperature. After three rinses with lysis buffer, the beads were heated and utilized for western blot which were conducted a minimum of three times for each experiment.

### 2.4 RNA extraction, reverse transcription and quantitative reverse transcription‒polymerase chain reaction

RNA isolation was carried out using TRIzol (Invitrogen), followed by cDNA synthesis using the PrimeScript RT Reagent Kit. Subsequent polymerase chain reaction (PCR) was done using SYBR (TaKaRa) on a reverse transcription‐PCR (RT‐PCR) System (Biosystems). The amplification process involved 40 PCR cycles consisting of an initial denaturation phase consisting of 95°C for 5 s and 60°C for 30 s in each cycle. Target gene expression was standardized against β‐actin or GAPDH levels. PCR primer details can be found in Table S2.

### 2.5 Immunohistochemical analysis

Antigen retrieval was performed for 20 min in ethylenediaminetetraacetic acid‐Tris buffer, followed by incubation of the paraffin‐embedded sections with antibodies targeting TRIM21 (#12108‐1‐AP, Proteintech), Vimentin (#5741, CST), CCT6A (#ab110905, Abcam), E‐cadherin (#3195, CST), Ki‐67 (#GB121141, Servicebio), CD4 (#GB15064, Servicebio), GATA3 (#5852, CST), T‐bet (#97135, CST) and FOXP3 (#GB112325, Servicebio). After washing, the sections were exposed to a biotinylated secondary antibody from Kirkegaard & Perry Laboratories. A semi‐quantitative evaluation was performed based on the Fromowitz criteria,^20^ with two independent pathologists conducting the assessment without access to patient details.

### 2.6 Immunofluorescence

Cells were seeded on 10‐mm coverslips in 6‐well plates and left to incubate for 24 h. Subsequently, they were washed with 1× PBS and then treated with 4% paraformaldehyde for fixation. The cells on coverslips or within paraffin‐embedded sections were exposed to antibodies (targeting Slug (#9585, CST), E‐cadherin (#3195, CST), IFN‐γ (#15365‐1‐AP, Proteintech), CD8 (#GB15068, Servicebio), CD4 (#GB15064, Servicebio), FOXP3 (#GB112325, Servicebio) GATA3 (#5852, CST) and T‐bet (#97135, CST)) overnight at 4°C. Following this, cells on coverslips underwent incubation with a DAPI and fluorescent dye‐conjugated secondary antibody before examination via fluorescence microscopy.

### 2.7 Cell function assays

Cell proliferation was evaluated using a cell counting kit‐8 assay (#CK04, Dojindo). TNBC cells were placed in 96‐well plates and cultured for 0, 24, 48, 72, or 96 h. At each interval, 100 µL reagent was added. Incubation of the cells was conducted at 37°C for 2 h, followed by measuring OD450 with a microplate reader (Bio‐Rad). For migration and invasion assays, TNBC cells cultured without FBS overnight were seeded into the upper compartments of uncoated Transwell plates (to assess migration) or Matrigel‐coated Transwell plates (to assess invasion) (#3422, Corning). After 24–48 h of incubation, the cells were treated with methanol for fixation, and then crystal violet (Beyotime) was used for staining, and counted in three random fields on the lower surface of each membrane. Each experiment was conducted thrice for consistency.

### 2.8 Flow cytometry assays

For apoptosis analysis, cells were collected and evaluated via the Apoptosis Kit (#88‐8007‐74, eBioscience) 48 h after transfection. Cell cycle assessments were conducted utilizing the Cell cycle and cell apoptosis detection Kit (#C1052, Beyotime) following the provided guidelines. In the context of tumour immune microenvironment (TIME) investigation, subcutaneous tumours were sectioned into 2‐mm^3^ fragments. These tissues underwent digestion and were treated using collagenase D for 30 min. Following this, all cells were washed in PBS supplemented with 2% FBS. The cells were then treated with the Zombie AquaTM Fixable Viability Kit (#423101, Biolegend) and antibodies targeting CD45 (#103115, Biolegend), CD4 (#116013, Biolegend), CD8a (#100733, Biolegend), PD1 (#135217, Biolegend) and CTLA4 (#106305, Biolegend). All specimens were stained and analyzed using an LSRFortessa (BD), with data interpretation carried out using FlowJo software (TreeStar).

### 2.9 RNA sequencing

Each MDA‐MB‐231 cell sample yielded 1 µg of RNA. Subsequently, the clean reads underwent alignment to the reference genome employing the HISAT2 spliced read aligner from the Ensembl Human Genome Assembly for analysis. Gene expression levels were quantified in RPKM values. The differentially expressed genes were assessed for KEGG pathway enrichment using the DAVID tool available at https://david.ncifcrf.gov/.

### 2.10 Liquid chromatography‐tandem MS analysis

Tryptic peptides, dissolved in 0.1% formic acid (termed buffer A), were loaded onto a dedicated reversed‐phase analytical column. The gradient involved transitioning from 6% to 23% buffer B (0.1% formic acid in 98% acetonitrile) within 16 min, followed by a transition from 23% to 35% solvent B across 8 min, a further escalation to 80% over 3 min, and maintenance at 80% for the final 3 min. Throughout these procedures, a consistent flow rate of 400 nL/min was maintained. Subsequently, the peptides underwent NSI–tandem MS (MS/MS). Select peptide with NCE 28 for MS/MS analysis, identifying fragments at a resolution of 17500 in the Orbitrap. Employing data‐dependent methods, alternating between 1 MS scan and 20 MS/MS scans, with a dynamic exclusion time of 15.0 s.

### 2.11 Animal experiments

Note that, 4–5‐week female athymic nude and BALB/c mice, free of pathogens, were procured from Shanghai Model Organisms. They were housed in a specific pathogen‐free environment tailored for mice for a week before commencing injections to allow them to acclimate. For the experiments, either 100 µL of PBS containing 1 × 10^5^ 4T1 cells or their variants were then administered via the tail veins of nude mice. Meanwhile, MDA‐MB‐231 cells (1 × 10^6^), 4T1 cells (5 × 10^5^), or their derivatives were added to 100 µL of PBS and introduced into the mammary fat pads of the mice as per a predefined protocol (with 5–7 mice in each group). Mice were humanely euthanized on Day 25 after the tail vein injection or Day 28 following the mammary fat pad administration. Tumour growth was tracked with regular calliper measurements, and tumour volumes were computed: (width^2^ × length)/2. Metastatic sites were detected utilizing BLI. Regarding in vivo drug interventions, starting 7 days post‐inoculation, the tumour‐bearing mice received Ipatasertib (30 mg/kg/day) via irrigation (i.g.) and/or anti‐PD1 (12.5 mg/kg/biw) via intraperitoneal (i.p.) injection. All animal procedures adhered to the ethical guidelines set by the Animal Care Committee at FUSCC.

### 2.12 Silver staining

The instructions for the use of silver staining can be found in the manual (#P0017S, Beyotime)

### 2.13 Survival analysis

RNA‐sequencing (RNA‐seq) information sourced from The Cancer Genome Atlas (https://cancergenome.nih.gov/). To evaluate gene expression and survival patterns, Breast Cancer Gene‐Expression Miner v5.1 (bc‐GenExMiner v5.1) (https://bcgenex.ico.unicancer.fr/) and KM‐Plotter (https://kmplot.com) were utilized. While bc‐GenExMiner v5.1 leverages curated breast cancer transcriptomic data from DNA microarrays and RNA‐seq, KM‐Plotter examines the relationship between gene expression levels and survival results across various tumour categories.

### 2.14 Statistical analysis

SPSS 20.0 and GraphPad Prism 9 software were used for statistical analysis. The relationship between CCT6A expression and TNBC patients' clinicopathologic parameters was evaluated through Fisher's exact test (two‐sided) or the chi‐square test. TRIM21 expression's correlation with survival in TNBC patients was evaluated utilizing the Kaplan‐Meier method alongside the log‐rank test. Group data significance was determined through one‐way analysis of variance or unpaired two‐tailed Student's *t*‐test. *p* < .05 denotes significant statistical significance. (****p < .0001, ***p < .001, *p < .01 and *p < .05; ns: not significant).

## 3 RESULTS

### 3.1 CCT6A is an independent prognostic factor for TNBC

**FIGURE 1:**
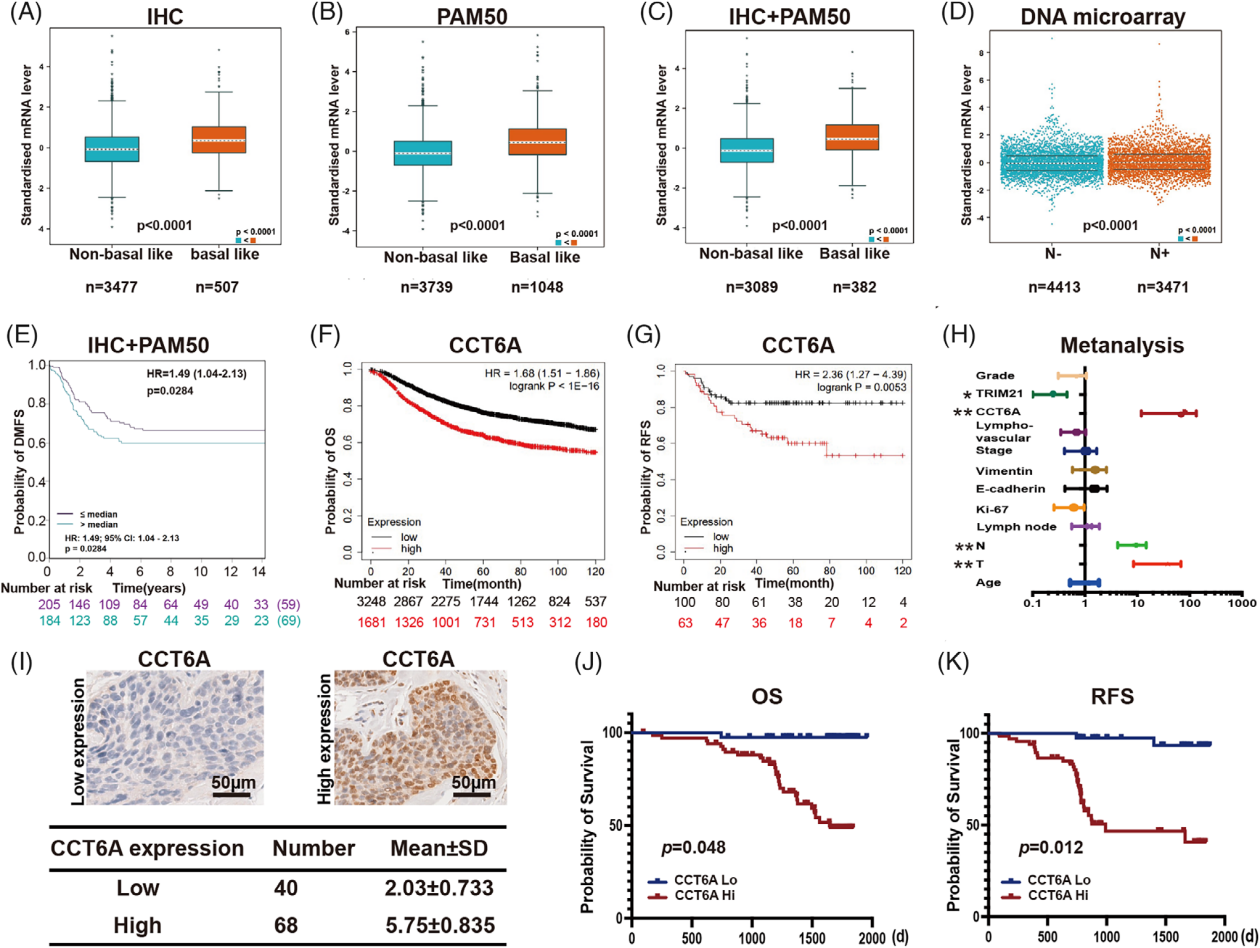
Chaperonin‐containing TCP1 subunit 6A (CCT6A) is an independent prognostic factor for triple‐negative breast cancer (TNBC). (A) Expression of CCT6A in non‐TNBC (*n* = 3477) and TNBC (*n* = 507) samples, as evaluated by Immunohistochemical (IHC) staining. (B) Expression of CCT6A in non‐basal‐like (*n* = 3739) and basal‐like (*n* = 1048) breast cancer tissues determined by PAM50 subtyping. (C) Expression of CCT6A in non‐basal‐like (*n* = 3089) and basal‐like (*n* = 382) breast cancer tissues, as assessed by PAM50 subtyping and IHC staining. (D) CCT6A expression in TNBC patients with positive lymph node metastasis. (E) Survival analysis of TNBC patients with high (*n* = 205) versus low (*n* = 184) CCT6A expression. (F) Kaplan–Meier analysis of overall survival (OS) in TNBC patients with high (*n* = 3248) versus low (*n* = 1681) CCT6A expression via KM‐plotter. (G) Kaplan–Meier analysis of RFS in TNBC patients with high (*n* = 100) versus low (*n* = 63) CCT6A expression via KM‐plotter. (H) Univariate analysis of the factors associated with OS in 108 TNBC patients. (I) The expression of CCT6A in TNBC clinical patients. (J) OS analysis of our 108 enrolled TNBC patients. (K) RFS analysis of our 108 enrolled TNBC patients.

To explore the expression of CCT6A in TNBC, Bc‐GenExMiner v5.1 was used to find that CCT6A was highly expressed in TNBC, according to Immunohistochemical (IHC) analysis (Figure 1A) and PAM50 subtyping (Figure 1B). The IHC and PAM50 results were verified by additional analyses (Figure 1C). TNBC cells exhibited high metastatic potential, and CCT6A was further found to be expressed at relatively high levels in TNBC patients with lymph node metastasis (Figure 1D). Survival analysis revealed that high expression of CCT6A in TNBC tissues was associated with shorter distant metastasis‐free survival (DMFS) times in patients (*p *= .028, hazard ratio [HR] = 1.49, 95% confidence interval [95%CI]: 1.04–2.13; Figure 1E). KM‐plotter analysis also indicated patients with high CCT6A expression had shorter overall survival (OS; log‐rank *P* < 1E‐16, HR: 1.68, 95%CI: 1.51–1.86, Figure 1F) and recurrence‐free survival (RFS; log‐rank *P* = .0053, HR: 2.36, 95%CI: 1.27–4.39; Figure 1G) times. Moreover, CCT6A expression showed significant associations with clinicopathologic factors such as N stage (*p* = .009), Vimentin level (*p* = .034) and Ki‐67 level (*p* = .036) as outlined in Table 1. Univariate analysis revealed associations between N stage (*p* = .001), T stage (*p* = .004), TRIM21 expression (*p* = .020), CCT6A expression (*p* = .006) and OS (Figure 1H). In the multivariate analysis, TRIM21 expression was deemed an independent prognostic factor for OS as indicated in Table S3. Furthermore, we evaluated CCT6A expression in our 108 enrolled TNBC samples, and immunohistochemical analysis revealed that the score for lower levels of CCT6A protein staining was 2.03 ± 0.733 for primary TNBC tissues and that for high levels of CCT6A protein staining was 5.75 ± 0.835 (Figure 1I). IHC staining demonstrated a notable elevation in CCT6A expression among TNBC patients with unfavourable OS (*p *= .048; Figure 1J) and RFS (*p *= .012; Figure 1K). This finding supported that CCT6A expression could serve as an autonomous prognostic indicator for TNBC.

**TABLE 1:** Correlation between different expression levels of TRIM21 and chaperonin‐containing TCP1 subunit 6A (CCT6A) with clinicopathological parameters of individuals with triple‐negative breast cancer (TNBC).

		TRIM21			CCT6A		
Clinicopathological parameters	Number	Low (*n* = 72)	High (*n* = 36)	*p*	Low (*n* = 40)	High (*n* = 68)	*p*
**age (median)**				1			0.842
≤51	54	36	18		21	33	
>51	54	36	18		19	35	
**Tumour size (cm)**				0.844			0.515
T ≥ 2	42	27	15		16	26	
2 < T ≤ 5	61	42	19		21	40	
T > 5	5	3	2		3	2	
**T stage**				1			0.357
T1+T2	103	69	34		37	66	
T3+T4	5	3	2		3	2	
**N stage**				0.015			0.009
N0	60	34	26		29	31	
N1+N2	48	38	10		11	37	
**AJCC stage (8th)**				0.464			0.33
I+II	85	55	30		34	51	
III	23	17	6		6	17	
**Lymph node metastases**				0.834			0.315
No	66	43	23		27	39	
Yes	42	29	13		13	29	
**E‐Cadherin**				<0.0001			0.073
Low	79	62	17		25	54	
High	29	10	19		15	14	
**Vimentin**				0.23			0.034
Low	25	14	11		14	11	
High	83	58	25		26	57	
**Ki‐67**				1			0.036
< 50	37	25	12		19	18	
> 50	71	47	24		21	50	
**Lympho‐vascular invasion**				1			0.416
No	67	45	22		27	40	
Yes	41	27	14		13	28	
**Grade**				1			0.812
Low	24	16	8		8	16	
High	84	56	28		32	52	

### 3.2 CCT6A facilitates proliferation, suppresses apoptosis and G1/S arrest in TNBC cells

**FIGURE 2:**
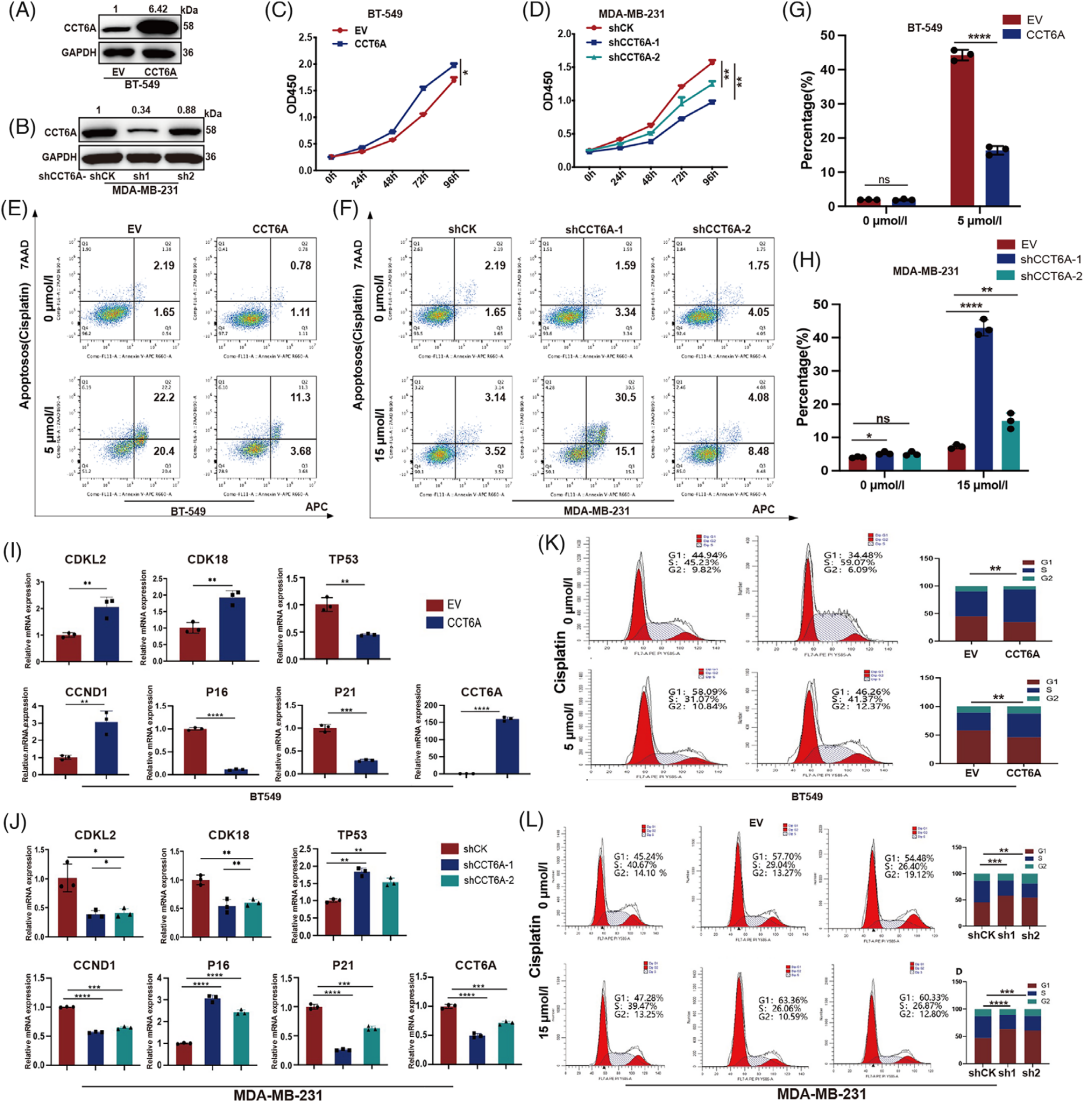
Chaperonin‐containing TCP1 subunit 6A (CCT6A) promotes the proliferation and inhibits the apoptosis of triple‐negative breast cancer (TNBC) cells. Expression of CCT6A in BT‐549 cells overexpressing CCT6A or EV control (A) and in MDA‐MB‐231 cells expressing shCK (control) or CCT6A shRNAs (B). The proliferation of the indicated BT‐549 (C) and MDA‐MB‐231 (D) cells was evaluated via a CCK‐8 assay. Apoptosis in the indicated BT‐549 (E) and MDA‐MB‐231 (F) cells was evaluated via FACS. (G) Statistical graph of the data in (E). (H) Statistical graph of the data in (F). qPCR was used to assess the expression of cell cycle‐related genes in the indicated BT‐549 (I) and MDA‐MB‐231 (J) cells. FACs were used to assess the cell cycle in the indicated BT‐549 (K) and MDA‐MB‐231 (L) cells. Each experiment was repeated three times.

We constructed a stably transduced BT‐549 cell line with CCT6A overexpression (Figure 2A) and a stably transduced MDA‐MB‐231 cell line with CCT6A knockdown (Figure 2B). CCT6A overexpression enhanced BT‐549 cell proliferation (Figure 2C), whereas CCT6A knockdown suppressed proliferation in MDA‐MB‐231 (Figure 2D). Additionally, the FACs assay revealed that CCT6A overexpression attenuated Cisplatin‐induced apoptosis in BT‐549 cells (Figure 2E,G), whereas CCT6A knockdown increased apoptosis in MDA‐MB‐231 cells (Figure 2F,H). BAX and BCL‐2 are essential markers for apoptosis. Consequently, quantitative PCR (qPCR) and western blots were conducted to delve deeper into the involvement of CCT6A in apoptosis. Our results revealed that in BT549^CCT6A^ cells, the expression of pro‐apoptotic protein BAX was elevated, whereas that of the apoptosis suppressor BCL‐2 was reduced (Figure S1A,C). Conversely, in the MDA‐MB‐231^shCCT6A^ cell line, BAX levels decreased and BCL‐2 levels increased (Figure S1B,D). Subsequent investigations explored the impact of CCT6A on the cell cycle. Our results revealed that CCT6A overexpression upregulated the expression of cell cycle‐related genes such as CDKL2, CDK18 and CCND1, while concurrently downregulating the expression of TP53, P16 and P21. Notably, cell cycle assays confirmed that CCT6A overexpression inhibited G1/S phase arrest. Overall, these findings suggested that CCT6A promoted proliferation, suppressed apoptosis and G1/S phase arrest in TNBC cells.

### 3.3 CCT6A promotes metastasis in TNBC cells

**FIGURE 3:**
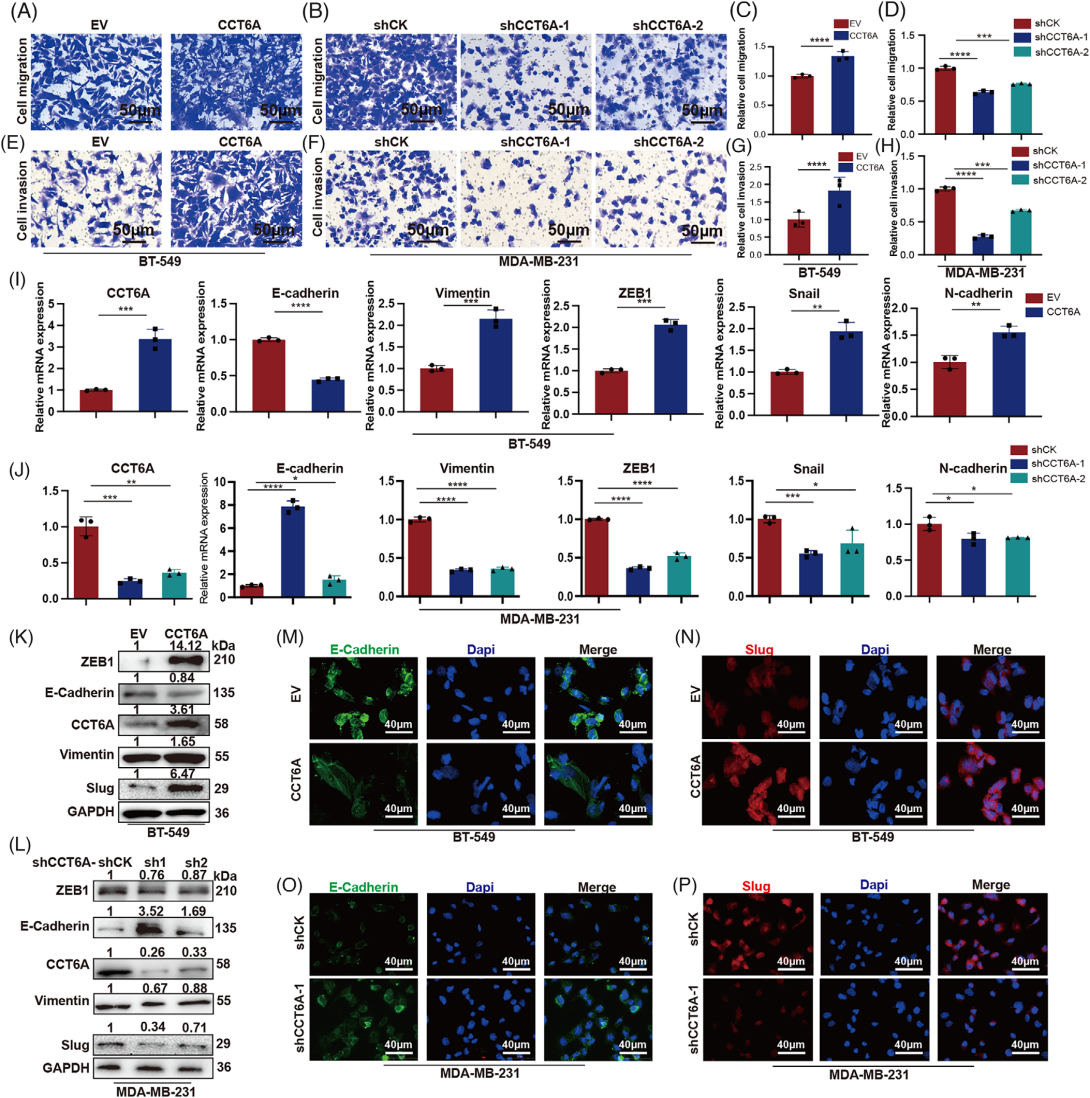
Chaperonin‐containing TCP1 subunit 6A (CCT6A) promotes metastasis in triple‐negative breast cancer (TNBC) cells. Migration of BT‐549 cells (A) overexpressing CCT6A or EV control and MDA‐MB‐231 cells (B) treated with shCK (control) or shRNAs as determined via a Transwell assay. (C, D) Quantification of the numbers of migrated cells in (A) and (B). Invasion of BT‐549 cells (E) overexpressing CCT6A or EV control and MDA‐MB‐231 cells (F) treated with shCK or shRNAs as determined via a Transwell assay. (G, H) Quantification of the numbers of invaded cells in (E) and (F). Epithelial‐mesenchymal transition (EMT) marker expression was analyzed by qPCR in BT‐549 cells (I) overexpressing CCT6A or the EV control and in MDA‐MB‐231 cells (J) treated with shCK or shRNAs. EMT marker expression was analyzed by western blotting in BT‐549 cells (K) expressing CCT6A or EV control and in MDA‐MB‐231 cells (L) treated with shCK or shRNAs. Immunofluorescence images showing the expression of the epithelial marker CDH1 (E‐cadherin) (M) and the mesenchymal marker Slug (N) in the indicated CCT6A‐overexpressing cells. Immunofluorescence images showing the expression of E‐cadherin (O) and Slug (P) in the indicated CCT6A‐knockdown cell lines. Each experiment was repeated three times. IB, immunoblot.

Given that CCT6A is highly expressed in lymph node metastasis patients, we sought to determine whether CCT6A affects cell migration and invasion. Transwell assays revealed that overexpression of CCT6A significantly promoted BT‐549 cell migration (Figure 3A,C) and invasion (Figure 3E,G), whereas knockdown of CCT6A suppressed MDA‐MB‐231 cell migration (Figure 3B,D) and invasion (Figure 3F,H). EMT is crucial in metastasis; thus, we evaluated the expression of EMT markers in the indicated TNBC cells. In TNBC cell lines, overexpression of CCT6A significantly promoted EMT, increased the expression of Snail, Vimentin and ZEB1; and inhibited the expression of E‐Cadherin at the transcriptional (Figure 3I) and translational (Figure 3K) levels. On the other hand, the knockdown of CCT6A significantly inhibited EMT, increased the expression of Vimentin, Snail and ZEB1; and decreased E‐cadherin expression at the transcriptional (Figure 3J) and translational (Figure 3L) levels. Immunofluorescence assays indicated that ectopic CCT6A expression downregulated E‐cadherin expression (Figure 3M) and upregulated Slug expression (Figure 3N), whereas CCT6A knockdown obviously increased E‐cadherin expression (Figure 3O) and decreased Slug expression (Figure 3P) in TNBC cells. Therefore, these results indicated that CCT6A might induce EMT in TNBC.

### 3.4 TRIM21 destabilizes CCT6A through K48‐linked polyubiquitination

**FIGURE 4:**
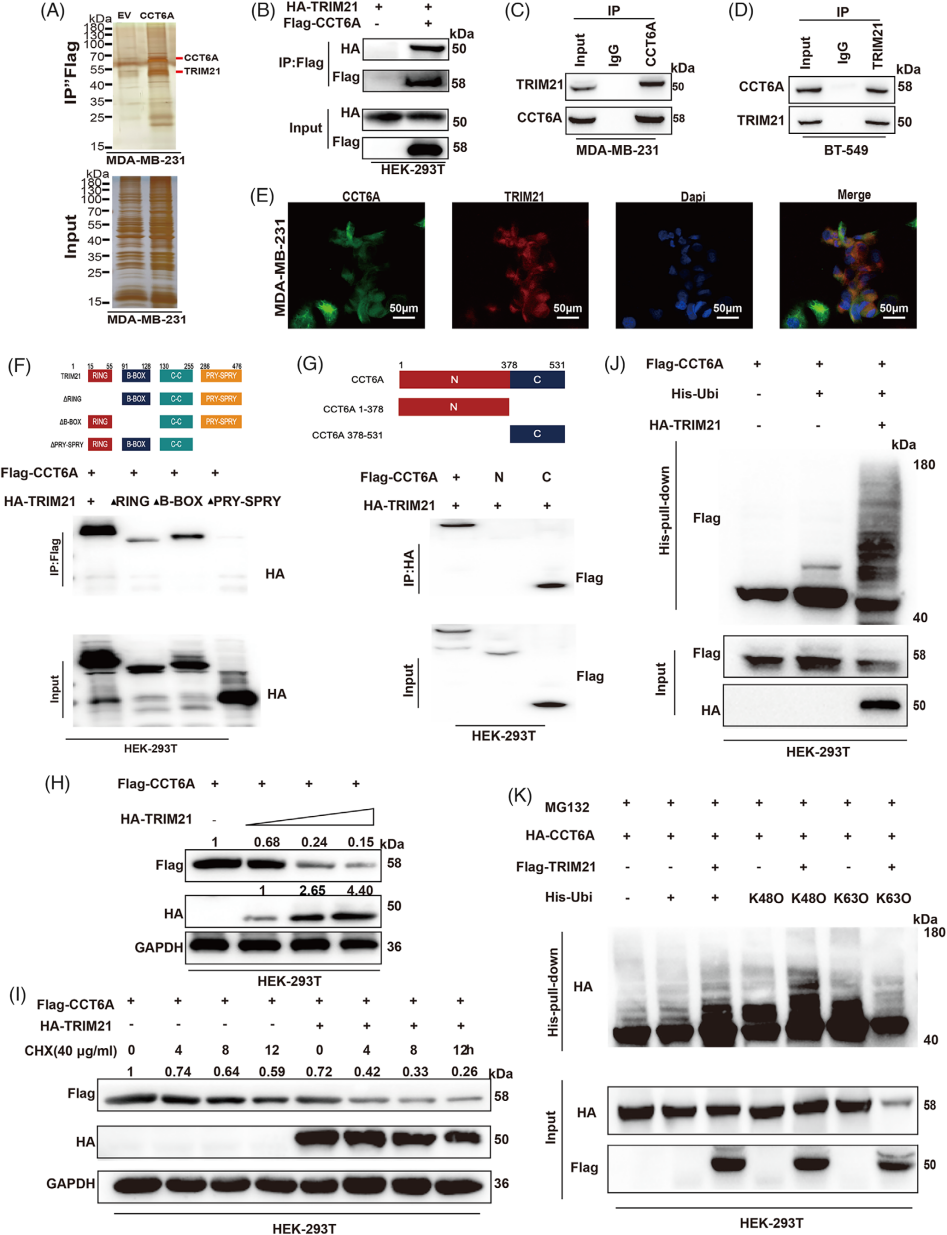
TRIM21 destabilizes Chaperonin‐containing TCP1 subunit 6A (CCT6A) through K48‐linked polyubiquitination. (A) Immunoprecipitation assay followed by silver staining of the CCT6A‐binding proteins. (B) Immunoprecipitation and western blot analysis of the interaction of Flag‐CCT6A with HA‐TRIM21 in HEK‐293T cells (*n* = 3). (C) Co‐immunoprecipitation of endogenous TRIM21 with an anti‐CCT6A antibody in MDA‐MB‐231 cells. (D) Co‐immunoprecipitation of endogenous CCT6A with an anti‐TRIM21 antibody in BT‐549 cells (*n* = 3). (E) Immunofluorescence assay was performed on MDA‐MB‐231 cells with an anti‐CCT6A antibody (green), an anti‐TRIM21 antibody (red) and DAPI (blue). The above data were derived from more than three independent experiments. (F) Different domains of TRIM21 were cotransfected with CCT6A into HEK‐293T cells. The cell lysates were subjected to immunoprecipitation with an anti‐Flag antibody as described, and the immunoprecipitates were then analyzed by western blotting. (G) A panel of CCT6A expression constructs was cotransfected with TRIM21 into HEK‐293T cells, followed by IP and western blotting as described. (H) Immunoblot analysis of Flag‐CCT6A and HA‐TRIM21 in the lysates of HEK‐293T cells cotransfected with Flag‐CCT6A and various amounts of HA‐TRIM21. (I) Immunoblot analysis was performed to evaluate the half‐life of Flag‐CCT6A in HEK‐293T cells transfected with Flag‐CCT6A and HA‐TRIM21. (J) HEK‐293T cells were cotransfected with HA‐TRIM21, Flag‐CCT6A and His‐ubiquitin and were then treated with 20 µM MG132 for 4 h. A ubiquitin pulldown assay for Flag‐tagged CCT6A was carried out as described in the Materials and Methods section. (K) K48‐only and K63‐only ubiquitin plasmids were transfected alone or with the HA‐TRIM21 and Flag‐CCT6A plasmids into 293T cells for 48 h, after which the cells were harvested and subjected to IP with an anti‐His antibody.

To delve deeper into the regulators governing CCT6A expression, we conducted a co‐IP–mass spectrometry (Co‐IP MS) analysis using MDA‐MB‐231 cell lysates, where TRIM21 emerged as a significant hit (Figure 4A). Subsequent Co‐IP assays confirmed the physical interaction between TRIM21 and CCT6A (Figure 4B). This interaction was further validated in TNBC cells through an endogenous IP assay using anti‐CCT6A and anti‐TRIM21 antibodies (Figure 4C,D). Immunofluorescence assay revealed that CCT6A and TRIM21 were colocalized primarily within the cytoplasm of MDA‐MB‐231 cells (Figure 4E). Truncation studies of TRIM21 and CCT6A pinpointed the PRY‐SPRY domain of TRIM21 as crucial for binding to CCT6A, with TRIM21 predominantly binding to the CCT6A region spanning amino acids 378–531 (Figure 4F,G). Given the functional interplay between these proteins, we investigated whether TRIM21 modulates CCT6A stability. Co‐transfection of CCT6A with either TRIM21 or a control vector in HEK‐293T followed by western blot demonstrated the concentration‐dependent degradation of CCT6A with increasing TRIM21 levels (Figure 4H). Subsequent treatment of transfected HEK‐293T cells with the CHX revealed a significantly shortened half‐life of CCT6A in the presence of TRIM21 (Figure 4I). The reduction in CCT6A triggered by TRIM21 overexpression was counteracted by the proteasome inhibitor MG132 (Figure S2). Furthermore, co‐transfection of CCT6A‐expressing plasmids with TRIM21‐expressing plasmids in HEK‐293T cells led to a notable increase in the ubiquitination of CCT6A by TRIM21 (Figure 4J). Currently, Lys (K) 48 ‐ and K63‐linked ubiquitination are the most well‐characterized types of ubiquitination.^21^ Notably, our investigations of the type of polyubiquitin chain attached to CCT6A by TRIM21 revealed that TRIM21 triggered K48‐linked ubiquitination of CCT6A, which is recognized to enhance proteasome‐dependent degradation of specific proteins.

### 3.5 TRIM21 ubiquitinates CCT6A and inhibits TNBC progression

**FIGURE 5:**
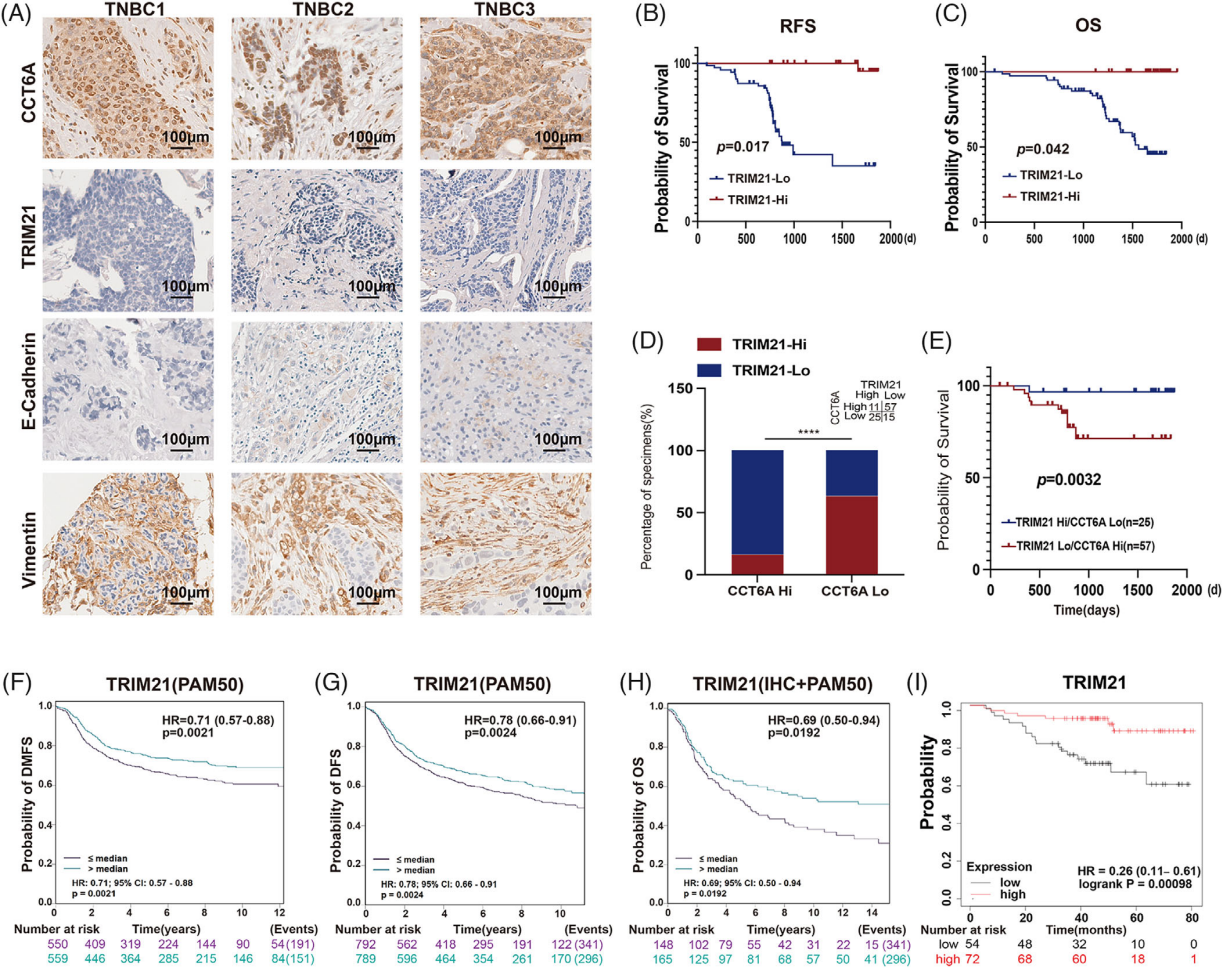
TRIM21 ubiquitinates Chaperonin‐containing TCP1 subunit 6A (CCT6A) and inhibits triple‐negative breast cancer (TNBC) progression. (A) Representative images of Immunohistochemical (IHC) staining for TRIM21, CCT6A, E‐cadherin and Vimentin in TNBC tissues (*n* = 3). (B) RFS analysis of our 108 enrolled TNBC patients. (C) Overall survival (OS) analysis of our 108 enrolled TNBC patients. (D) Correlation of the expression levels of TRIM21 and CCT6A in (A). (E) Comparison of the prognoses of TNBC patients with different patterns of endogenous TRIM21/CCT6A expression via Kaplan–Meier survival analysis. (F) Distant metastasis‐free survival (DMFS) analysis of the basal‐like breast cancer (PAM50) patients with low (*n* = 550) versus high (*n* = 559) TRIM21 expression. (G) DFS analysis of the basal‐like breast cancer patients (PAM50) with low (*n* = 191) versus high (*n* = 151) TRIM21 expression. (H) OS analysis of TNBC (IHC) and basal‐like breast cancer (PAM50) patients with low (*n* = 341) versus high (*n* = 296) TRIM21 expression. (I) RFS analysis of TNBC patients with low (*n* = 54) versus high (*n* = 72) TRIM21 expression via the KM‐plotter.

To investigate whether the ability of CCT6A to promote TNBC progression is affected by TRIM21‐mediated ubiquitination, we evaluated the TRIM21, E‐cadherin and Vimentin protein levels via IHC staining in a TNBC cohort comprising samples from 108 patients diagnosed at FUSCC (Table 1). We verified the relationships among TRIM21, CCT6A and EMT in clinical TNBC samples. It was observed that E‐cadherin expression was reduced while Vimentin expression was elevated in TNBC samples with TRIM21 low and CCT6A high (TRIM21^low^/CCT6A^high^) expression (Figure 5A). Our evaluation demonstrated that TNBC patients exhibiting elevated TRIM21 expression levels experienced notably improved RFS (*p *= .017) (Figure 5B) and OS (*p *= .042) (Figure 5C). Consistent with the expectations, a reverse correlation was observed between TRIM21 expression and CCT6A expression (*p *< .0001; Figure 5D). In addition, patients with TRIM21^low^/CCT6A^high^ expression had worse OS than those with other TRIM21/CCT6A expression patterns (*p *= .0032; Figure 5E). These findings further suggested that TRIM21 was related to CCT6A degradation. BC‐GenExMiner v5.1 revealed that TNBC patients with low TRIM21 expression had short DMFS (*p *= .0021, HR: 0.71, 95%CI: 0.57–0.88) (Figure 5F), DFS (*p *= .0024, HR: 0.78, 95%CI: 0.66–0.91) (Figure 5G), OS (HR: 0.69, 95%CI: 0.50–0.94, *p *= .0192) (Figure 5H) and RFS (log‐rank *P*: 0.00098, HR: 0.26, 95%CI: 0.11–0.61) (Figure 1I). It proved that high TRIM21 expression was associated with CCT6A degradation and favourable prognosis in TNBC patients.

### 3.6 TRIM21 ubiquitinates CCT6A and inhibits TNBC progression through its enzymatically active RING domain

**FIGURE 6:**
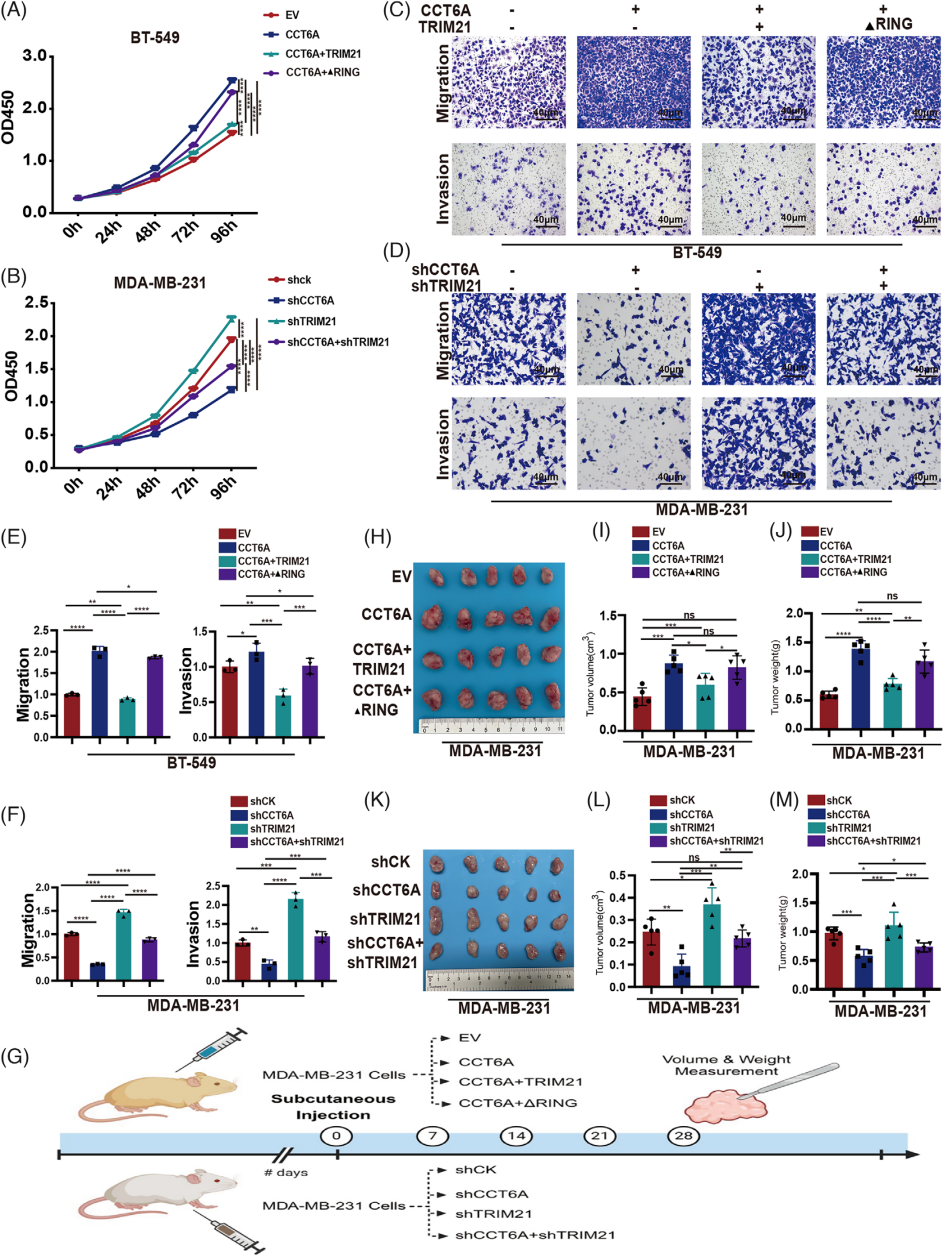
TRIM21 ubiquitinates Chaperonin‐containing TCP1 subunit 6A (CCT6A) and inhibits triple‐negative breast cancer (TNBC) progression through its enzymatically active RING domain. (A) Proliferation of BT‐549 cells expressing EV, CCT6A, CCT6A+TRIM21, or CCT6A+TRIM21‐ΔRING and (B) MDA‐MB‐231 cells expressing shCK (control), shCCT6A, shTRIM21 or shCCT6A+shTRIM21(B). Migration and invasion of the indicated BT‐549 (C) and indicated MDA‐MB‐231 cells (D). (E, F) Quantification of the numbers of migrated and invaded cells in (C) and (D). (G) Schematic overview of animal experimentation. (H) Representative morphology of subcutaneous tumours from nude mice bearing MDA‐MB‐231 cells transfected with EV, CCT6A, CCT6A+TRIM21 or CCT6A+ TRIM21‐ΔRING. The tumour volume (I) and tumour weight (J) were recorded after injecting the mice with the abovementioned stably transduced cells. (K) Representative morphology of subcutaneous tumours from nude mice bearing MDA‐MB‐231 cells transduced with shCK, shCCT6A, shTRIM21 or shCCT6A+shTRIM21. The tumour volume (L) and tumour weight (M) were recorded after injecting the mice with the abovementioned stably transduced cells.

To demonstrate that CCT6A promotes TNBC progression through TRIM21‐mediated ubiquitination, we generated CCT6A‐overexpressing BT‐549 cells that overexpress TRIM21 or a TRIM21 mutant with deletion of the RING domain (TRIM21‐ΔRING). Compared with BT‐549^CCT6A^ cells, BT‐549^TRIM21+CCT6A^ cells exhibited significantly reduced proliferation, migration and invasion capabilities. Compared with BT‐549^TRIM21+CCT6A^ cells, BT‐549^CCT6A+TRIM21‐ΔRING^ cells exhibited significantly increased proliferation, migration and invasion (Figure 6A,C,E) capabilities. Moreover, MDA‐MB‐231^shCCT6A^ inhibited cell proliferation, migration and invasion; however, MDA‐MB‐231^shCCT6A+shTRIM21^ partially abolished the suppression of cell proliferation, migration and invasion (Figure 6B,D,F) caused by MDA‐MB‐231^shCCT6A^. Therefore, we concluded that TRIM21 inhibited TNBC progression via its E3 ubiquitin ligase activity via its enzymatically active RING domain to promote the degradation of CCT6A in vitro.

We further verified the effects of TRIM21‐mediated ubiquitination and degradation of CCT6A on TNBC in vivo (Figure 6G). When MDA‐MB‐231^EV^, MDA‐MB‐231^CCT6A^, MDA‐MB‐231^CCT6A+TRIM21^ or MDA‐MB‐231^CCT6A+TRIM21‐ΔRING^ cells were injected into nude mice, compared with MDA‐MB‐231^CCT6A^ cells, MDA‐MB‐231^CCT6A+TRIM21^ cells presented significantly reduced tumour weight and tumour volume. Compared with the MDA‐MB‐231^CCT6A+TRIM21^ group, the MDA‐MB‐231^CCT6A+TRIM21‐ΔRING^ group presented significantly greater tumour volume and tumour weight (Figure 6H–J). Consistent with the expectations, the MDA‐MB‐231^shTRIM21^‐injected mice produced larger subcutaneous tumours, in terms of both weight and volume, than the MDA‐MB‐231^shCCT6A+shTRIM21^‐injected mice did (Figure 6K–M). These findings indicated that the knockdown of TRIM21 partially reversed the reduction in tumour size caused by the knockdown of CCT6A.

**FIGURE 7:**
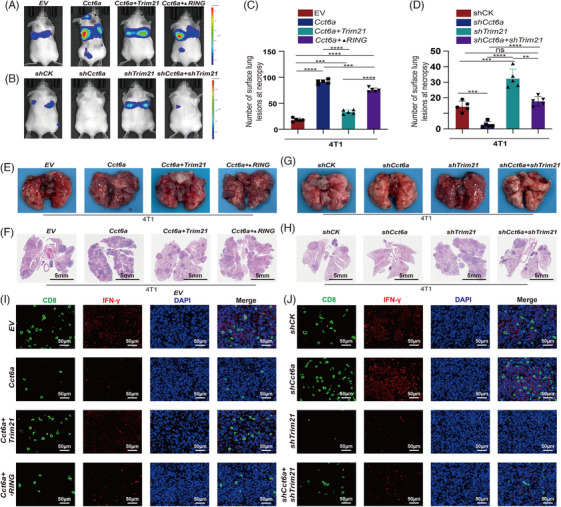
TRIM21 ubiquitinates Chaperonin‐containing TCP1 subunit 6A (CCT6A) and inhibits triple‐negative breast cancer (TNBC) metastasis through its enzymatically active RING domain. (A) Schematic overview of animal experimentation. (B) 4T1 cells expressing *EV* (control), *Cct6a, Cct6a+Trim21*, or *Cct6a+Trim21‐ΔRING* as indicated were injected into mice through the tail vein (n = 5). Metastasis was monitored (B), and representative bright‐field images (F) and H&E staining images (G) of the lung metastases in BALB/c mice bearing the indicated 4T1 cells were acquired. (D) The number of lung metastases was counted. 4T1 cells expressing *shCK* (control), *shCct6a, shTrim21*, or *shCct6a+shTrim21* as indicated were injected into mice through the tail vein (n = 5). Metastasis was monitored (C), and representative bright‐field images (H) and H&E staining images (I) of the lung metastases in BALB/c mice bearing the indicated 4T1 cells were acquired. (E) The number of lung metastases was counted. (J, K) CD8+T cells and IFN‐γ were stained by immunofluorescence in the indicated tumour samples.

Furthermore, we intravenously injected engineered luciferase‐labeled 4T1 cells to assess the influence of TRIM21 and CCT6A on cancer metastasis (Figure 7A). Both the number of metastatic sites and the maximum standard uptake value (SUVmax) were greater in the 4T1*^Cct6a^* cell‐injected mice than in the 4T1*^Cct6a+Trim21^* cell‐injected mice. Compared with the 4T1*^Cct6a+Trim21^* group, the 4T1*^Cct6a+Trim21‐ΔRING^* group presented significantly increased metastatic sites and SUVmax values (Figure 7B,D,F,G). Mice injected with 4T1*^shTrim21^* cells exhibited more metastatic sites and a greater SUVmax than mice injected with 4T1*^shCct6a^* ^+^ *^shTrim21^* cells or 4T1*^shCct6a^* cells, and the 4T1*^shTrim21^* group partially reversed the prometastatic effect caused by 4T1*^Cct6a^* (Figure 7C,E,H,I). In addition, TIME analysis revealed an increase in Treg cell count and FOXP3 expression in the lung metastatic tissues of 4T1^Cct6a^‐indicated mice, compared with those in the 4T1^EV^‐indicated mice. We also found a decrease observed in the tumours of the 4T1*^Cct6a+Trim21^* mouse group compared with those in the 4T1*^Cct6a+Trim21‐ΔRING^* mouse group (Figure 7J). Moreover, a reduction in CD8+ T/IFN‐γ expression (Figure 7K) and Th1/T‐bet expression was detected in the lung metastasis tissues of the mice in the 4T1*^Cct6a^* group compared with those in the 4T1^EV^ group, whereas a rise in tumour count in the 4T1*^Cct6a+Trim21^* mice group compared with those in the 4T1*^Cct6a+Trim21‐ΔRING^* mouse group was detected. Furthermore, there was a rise in the quantity of Th2/GATA3 expression in the lung metastasis tissues of mice injected with the 4T1*^Cct6a^* group compared to 4T1^EV^, showing a decline in the tumours of the 4T1*^Cct6a+Trim21^* mice group compared to the 4T1*^Cct6a+Trim21‐ΔRING^* mouse group (Figure S3A,C). Moreover, 4T1*^shCct6a^* increased the number of CD8+T/IFN‐γ expression and Th1/T‐bet expression, and decreased the number of Treg/Foxp3 and Th2/GATA3 cells expression; however, 4T1*^shCct6a+shTrim21^* partially reverse the changes in the immune microenvironment caused by 4T1*^Cct6a^* cells (Figure S3B,D). These findings indicated that TRIM21 mediated the degradation of CCT6A and inhibited TNBC progression through its E3 ubiquitin ligase RING domain both in vivo and in vitro.

### 3.7 Ipatasertib reverses the aggressive phenotype of CCT6A‐overexpressing cells

**FIGURE 8:**
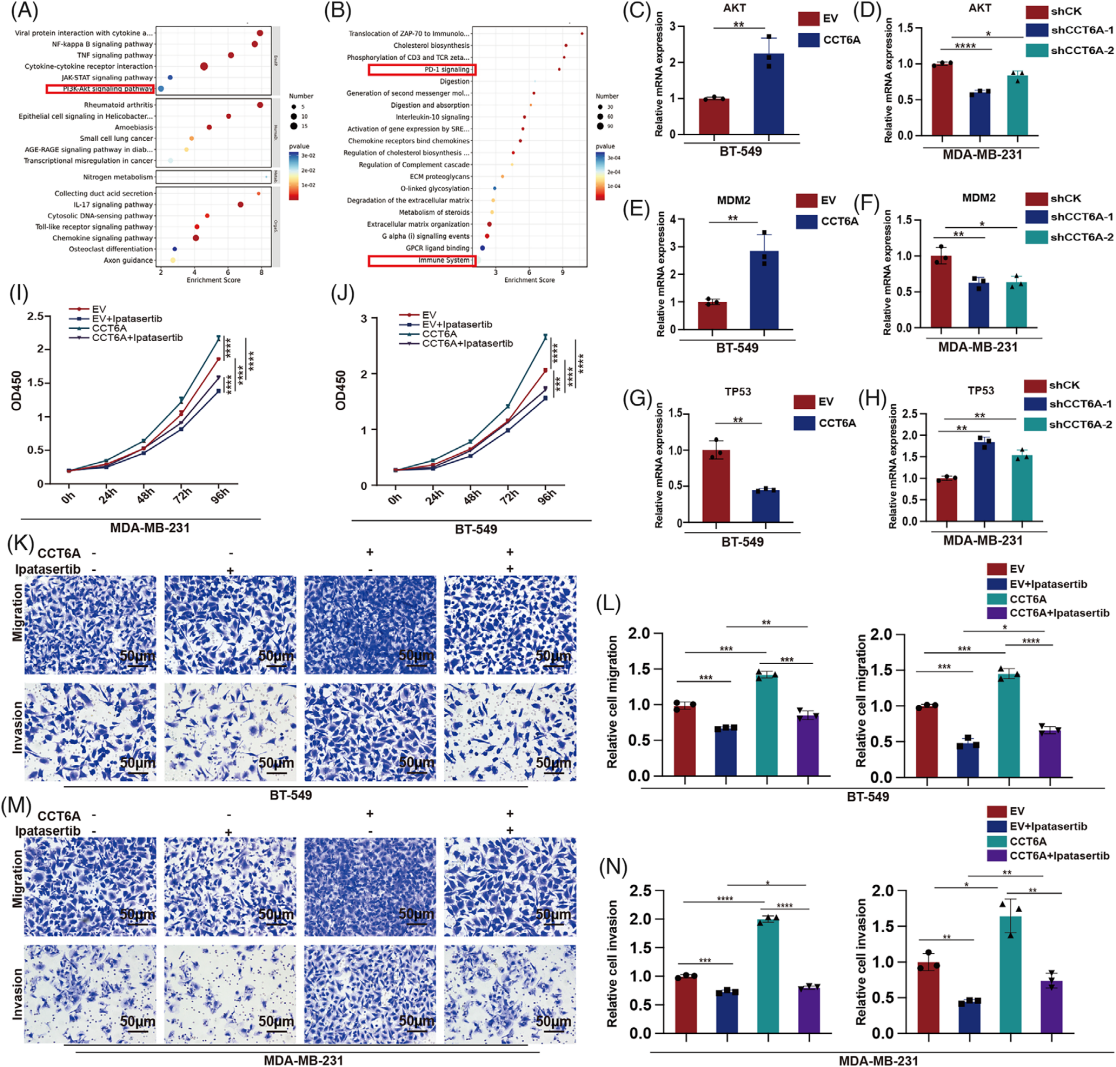
Ipatasertib reverses the aggressive phenotype of Chaperonin‐containing TCP1 subunit 6A (CCT6A)‐overexpressing cells. (A) KEGG analysis indicated that the genes downregulated by shCCT6A transduction were predominantly associated with the PI3K/AKT signalling pathway. (B) Reactome pathway analysis revealed that the genes downregulated by shCCT6A transduction were predominantly associated with PD1 signalling and the immune system. (C) AKT, (E) MDM2 and (G) TP53 expression were evaluated in BT‐549 cells expressing CCT6A or the EV control via qPCR. (D) AKT, (F) MDM2 and (H) TP53 expression were evaluated in MDA‐MB‐231 cells treated with shCK or shRNAs via qPCR. The cell proliferation of MDA‐MB‐231 (I) and BT‐549 (J) cells expressing EV or CCT6A and treated with or without Ipatasertib was evaluated via a CCK‐8 assay. (K, L) The migration and invasion of BT‐549 cells expressing EV or CCT6A and treated with or without ipatasertib were evaluated via Transwell assays. (M, N) The migration and invasion of MDA‐MB‐231 cells expressing EV or CCT6A and treated with or without Ipatasertib were evaluated via Transwell assays. Each experiment was repeated three times.

To understand how CCT6A enhances EMT, cell migration, invasion and proliferation, we conducted RNA‐seq to identify CCT6A's downstream targets. KEGG analysis revealed that proteins involved in the PI3K/AKT pathway were enriched in MDA‐MB‐231^CCT6A^ (Figure 8A). Reactome pathway analysis revealed that PD1 signalling and immune system pathways were enriched (Figure 8B). Previous studies have elucidated that AKT regulates TNBC cell EMT, motility and metastasis^22^ and that the AKT/MDM2/TP53 signalling pathway is important in TNBC.^23^ We further investigated the expression of the TRIM21/CCT6A/AKT signalling pathway in the TNBC tissue. We found that TRIM21 expression was decreased in TNBC, but highly expressed in para‐TNBC. CCT6A and p‐AKT were highly expressed in TNBC and expressed at low levels in para‐TNBC (Figure S4). qPCR was thus performed to determine the function of CCT6A in regulating AKT/MDM2/TP53 expression, and the findings indicated that upregulation of CCT6A enhanced AKT (Figure 8C) and MDM2 expression (Figure 8E) and decreased the expression of TP53 (Figure 8G). Knockdown of CCT6A decreased AKT (Figure 8D) and MDM2 (Figure 8F) expression and increased TP53 expression (Figure 8H). Western blot analysis further revealed that CCT6A activated the AKT signalling pathway by increasing the phosphorylation of AKT/mTOR and p‐P53 while decreasing P53 (Figure S5). Ipatasertib, a pharmacological inhibitor of AKT, partially reversed CCT6A overexpression‐induced cell proliferation, migration and invasion in BT‐549 (Figure 8J–L) and MDA‐MB‐231 (Figure 8I,M,N) cells. Our results indicated that Ipatasertib might be applied to treat TNBC patients with high CCT6A expression.

### 3.8 Ipatasertib and/or anti‐PD1 therapy reverses the aggressive phenotype of CCT6A‐overexpressing cells by reshaping the immune microenvironment

**FIGURE 9:**
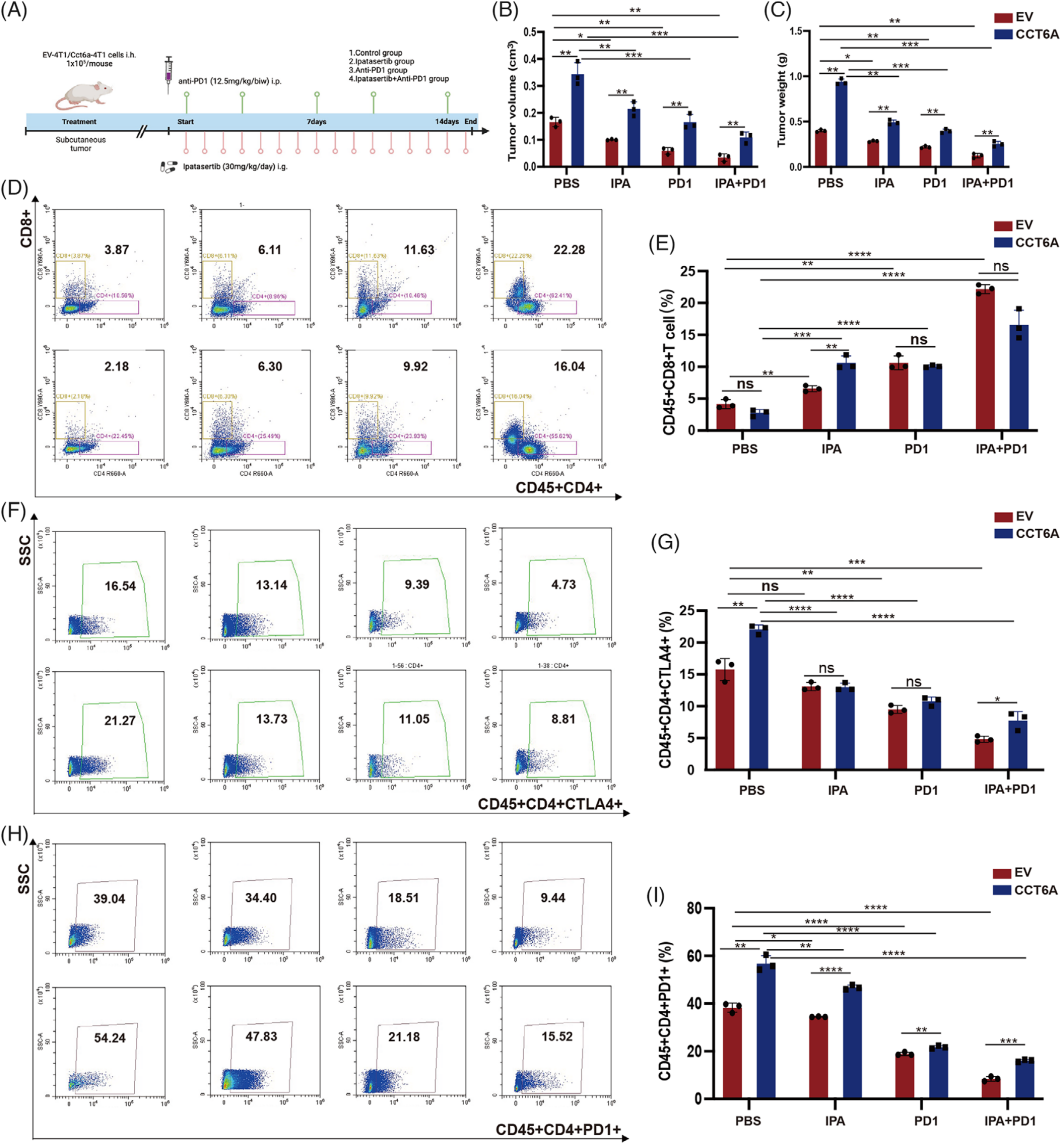
Reshaping the immune microenvironment to reverse the aggressive phenotype of Chaperonin‐containing TCP1 subunit 6A (CCT6A)‐overexpressing cells through Ipatasertib and/or Anti‐PD1 therapy. (A) Schematic overview of animal experimentation. After the introduction of the aforementioned stably transduced cells, tumour volume (B) and weight (C) were assessed post‐administration of Ipatasertib, Anti‐PD1 therapy, or the combination Ipatasertib with anti‐PD1 therapy. Flow cytometry analysis (FACS) was employed to evaluate the levels of CD45+CD8+ T cells (D, E), CD45+CD4+CTLA4+ T cells (F, G) and CD45+CD4+PD1+ T cells (H, I) following the aforementioned in vivo drug interventions.

To test the therapeutic effects of Ipatasertib and anti‐PD1 therapy in TNBC with high CCT6A expression, we injected 4T1^EV^ or 4T1*^Cct6a^* cells subcutaneously into C57BL/6 mice. On the seventh day post‐inoculation, the mice bearing tumours received Ipatasertib, anti‐PD1, or Ipatasertib combined with anti‐PD1 treatment for 15 days (Figure 9A). Ipatasertib, anti‐PD1, or Ipatasertib combined with anti‐PD1 significantly reduced the 4T1*^Cct6a^* group tumour volume (Figure 9B) and tumour weight (Figure 9C) compared with those of the 4T1^EV^ group. The tumour volume and weight after combination treatment were significantly lower than those after treatment with either Ipatasertib or anti‐PD1 therapy alone (Figure 9B,C). To further corroborate the impact of Ipatasertib and anti‐PD1 in an in vivo setting, we performed a TIME analysis on the mouse tissue samples from each treatment group. We found that Ipatasertib and anti‐PD1 therapy significantly increased the number of CD45+CD8+ T cells (Figure 9D,E), and decreased the number of CD45+CD4+CTLA4+ T cells (Figure 9F,G) and CD45+CD4+PD1+ T cells (Figure 9H,I). Collectively, these results confirmed that Ipatasertib and anti‐PD1 therapy suppressed tumourigenesis, probably in part by regulating signalling downstream of CCT6A and the TIME.

## 4 DISCUSSION

TNBC is characterized by high invasiveness, high metastatic ability and significant drug resistance.^24^ Although various drugs for TNBC are continuously emerging, their therapeutic efficacy in TNBC patients is still very limited.^25^ Research indicates that following neoadjuvant therapy, around two‐thirds of TNBC patients retain residual disease, placing them at a heightened risk of recurrence and metastasis, posing a significant clinical challenge for individuals with TNBC.^26^, ^27^ Therefore, studying the mechanism of TNBC is crucial for overcoming this major clinical problem.

This research noted a substantial increase in CCT6A expression linked to worse OS in TNBC patients. Furthermore, our findings highlighted the pivotal function of CCT6A in enhancing the growth, movement and infiltration of TNBC cells while suppressing their programmed cell death. Research has also shown that CCT6A plays a tumour‐promoting role in a variety of tumours, but the specific underlying mechanism remains unknown.^10^ For a deeper understanding of CCT6A's role in TNBC, we conducted co‐IP MS to unveil the proteins that interact with CCT6A. One of the candidates interacting proteins identified through this approach was TRIM21. Previous research has demonstrated that TRIM21 can facilitate HIF‐1α ubiquitination, leading to attenuation of renal cancer progression.^28^ We further examined the interaction and colocalization of TRIM21 with CCT6A. Moreover, we determined that the PRY‐SPRY domain of TRIM21 is bound to the region of CCT6A comprising aa 378–531. Subsequent analyses revealed that TRIM21 inhibited TNBC progression by promoting the degradation of CCT6A through its K48‐linked ubiquitination. This relationship was further validated in clinical TNBC samples, which presented low expression levels of TRIM21 and an inverse relationship was observed between TRIM21 expression and CCT6A expression. In vivo and in vitro tests further demonstrated that inactivation of the RING domain of TRIM21 E3 ligase activity in TNBC cells could inhibit K48‐linked CCT6A ubiquitination, thereby promoting TNBC proliferation, migration and invasion (Figure 7). TRIM21 was found to suppress the radiation‐induced dissemination of mitochondrial DNA and compromise the antitumour immune response in nasopharyngeal carcinoma.^29^ Furthermore, Li and colleagues highlighted CCT6A as a marker for the pre‐exhausted T‐cell subset in colorectal cancer.^30^ Our experiments in mice demonstrated a notably higher count of CD8+ T cells and an elevation in IFN‐γ secretion in the CCT6A/TRIM21 double‐overexpression cohort compared to the CCT6A group, and these effects were reversed in the corresponding knockdown and double‐knockdown groups.

RNA‐seq analysis has uncovered that the promotion of TNBC progression by CCT6A may be mediated through the AKT pathway. We discovered that Ipatasertib can oppose the cell migration, proliferation and invasion induced by CCT6A. The involvement of AKT signalling or EMT in TNBC lung metastasis has been extensively deliberated. Kumar et al. reported that targeting AKT signalling inhibited TNBC metastasis or EMT in TNBC lung metastasis.^31^ Tang et al. found that the disruption of FOXO1‐triggered LYPLAL1‐DT hindered TNBC advancement through the hnRNPK/β‐catenin complex.^32^ Our investigation unveiled a fresh pathway through which inhibiting CCT6A suppresses the progression of TNBC. Our research revealed a novel mechanism by which CCT6A inhibition suppresses TNBC progression. We found that TRIM21‐induced K48‐linked ubiquitination of CCT6A targets it for degradation, thereby inhibiting the AKT signalling pathway. The FAIRLANE clinical trial has underscored the necessity for further exploration of Ipatasertib in TNBC treatment.^33^

Simultaneously, the approval by the US Food and Drug Administration of the AKT inhibitor Capivasertib in conjunction with Fulvestrant for individuals with HR+/HER2‐ locally advanced or metastatic BC signified a notable achievement.^34^ Validation through in vivo drug administration experiments has highlighted the substantial reduction in CCT6A‐driven tumour growth achieved through the synergistic application of Ipatasertib and anti‐PD1 therapy. This discovery highlights a previously unidentified role for CCT6A in TNBC and suggests that targeting the TRIM21/CCT6A/AKT axis could be a therapeutic avenue to address BC. Our study not only establishes a theoretical framework for utilizing AKT inhibitors in TNBC therapy but also advocates for the combined use of AKT inhibitors and immunotherapy in countering CCT6A/AKT hyperactivity‐driven TNBC. Subsequent investigations will concentrate on assessing the viability of merging CCT6A inhibitors with additional targeted treatments to prolong survival for TNBC patients.

Our study is the first to discover that CCT6A is ubiquitinated by TRIM21. This finding opens new avenues for targeting protein modifications in breast cancer therapy. We have also underscored our novel discovery of the role of CCT6A in regulating p53, providing fresh perspectives for p53‐related research. However, one of the limitations was the restricted sample size for enrollment. Another limitation is that the effect of CCT6A on the tumour microenvironment in a mechanistic study is still at the phenotypic level. To address these problems, we will recruit more eligible patients and further explore the effect of CCT6A on the tumour microenvironment. In essence, our findings delineate how diminished TRIM21 expression in TNBC leads to reduced K48‐linked ubiquitination and subsequently increased expression of CCT6A, thus fostering TNBC progression via AKT pathway activation. These discoveries increase our understanding of the TRIM21/CCT6A/AKT axis in TNBC development, offering valuable insights for assessing the therapeutic potential of Ipatasertib and/or anti‐PD1, particularly in CCT6A‐overexpressing TNBC patients (see Graphical Abstract).

## AUTHOR CONTRIBUTIONS

Mengdi Yang: Data curation, formal analysis, investigation, methodology, writing original draft, supervision and funding acquisition. Jianing Cao: Writing original draft, investigation, formal analysis and validation. Shuangyue Pan: Investigation. Duancheng Guo and Tiantian Liu: Resources. Bin Li and Jinyan Wang: Software and visualization. Zhonghua Tao and Xichun Hu: Funding acquisition and project administration.

## CONFLICT OF INTEREST STATEMENT

The authors declare no conflict of interest.

## DATA AVAILABLE STATEMENT

Datasets and other files generated, analyzed, or processed in this study are available upon request from the corresponding author.

## ETHICS STATEMENT

This study was performed in accordance with the 1975 Declaration of Helsinki. This study was approved by the institutional ethics review board of Fudan University Shanghai Cancer Center and by the Institutional Animal Care and Use Committee of Fudan University Shanghai Cancer Center.

## Supporting information



Supporting Information
