# Supplementary material for: Chaperonin‐containing TCP1 subunit 6A inhibition via TRIM21‐mediated K48‐linked ubiquitination suppresses triple‐negative breast cancer progression through the AKT signalling pathway
Source: Clin Transl Med. 2024 Nov 18;14(11):e70097. doi: 10.1002/ctm2.70097 (PMC11571564; doi:10.1002/ctm2.70097)
Supplement: Supplementary file 1 — Supporting Information [file CTM2-14-e70097-s001.docx]

**Supplementary Figure legends**

**
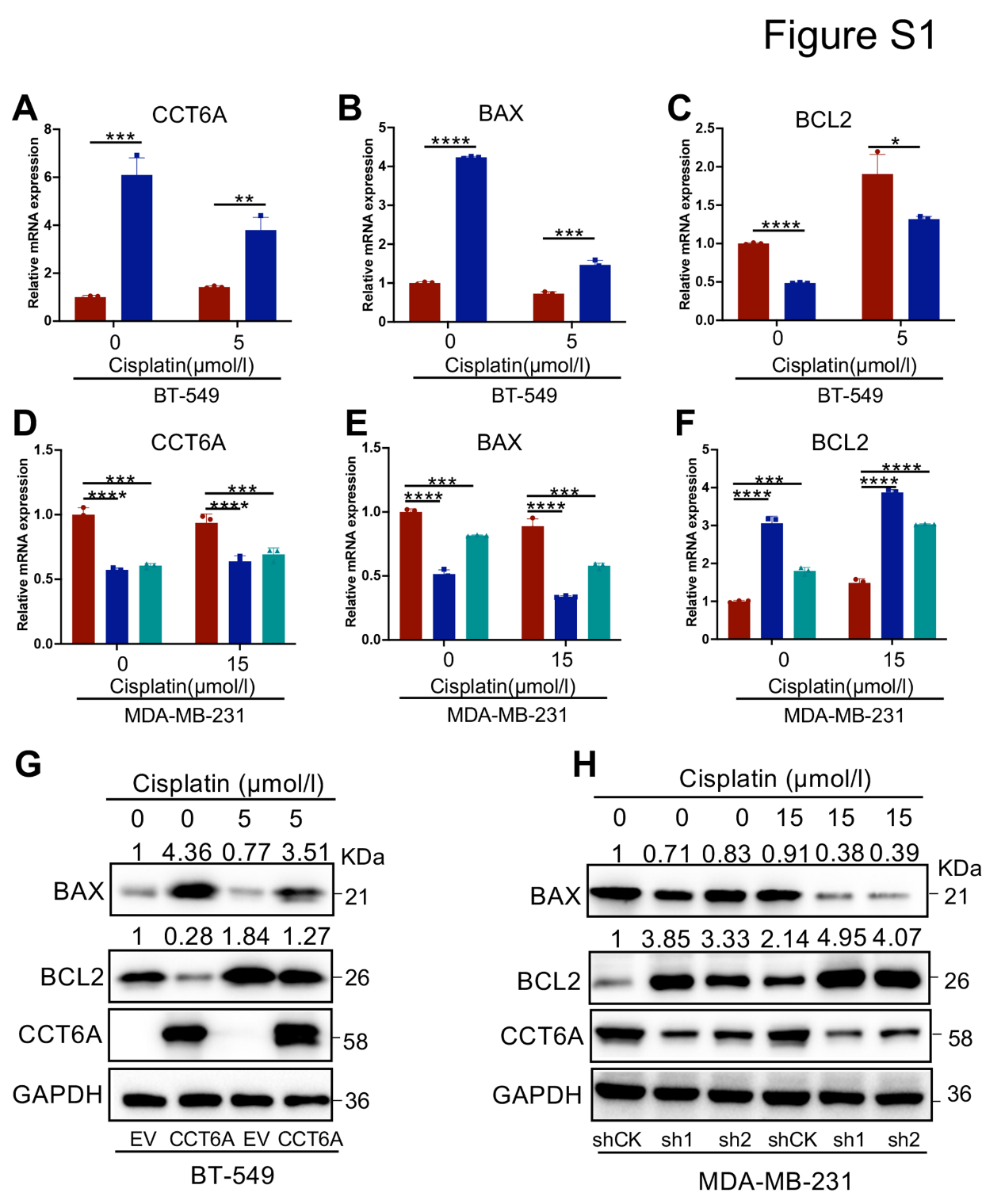
**

**Figure S1. CCT6A inhibits the apoptosis of TNBC cells**

qPCR was used to check cell apoptosis genes in indicated BT-549 (A) and MDA-MB-231 (B) cells. Western blot was used to check cell apoptosis genes in indicated BT-549 (C) and MDA-MB-231 (D) cells. Each experiment was repeated three times.

**
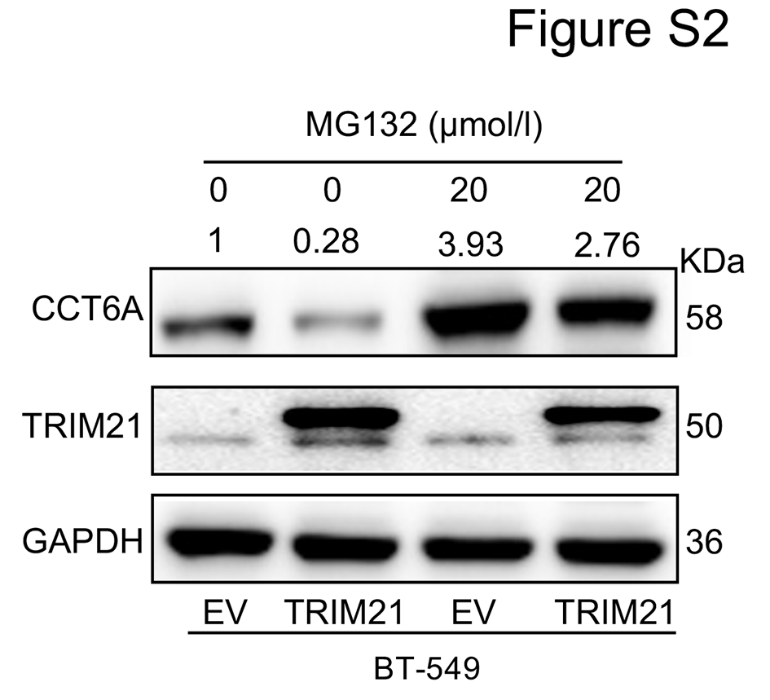
**

**Figure S2. The proteasome inhibitor MG132 inhibited the decrease in CCT6A induced by TRIM21 overexpression**

Western blot showing effects of the proteasome inhibitor MG132 (20 μmol/l for 6 h) treatment on CCT6A protein accumulation in BT-549.

**
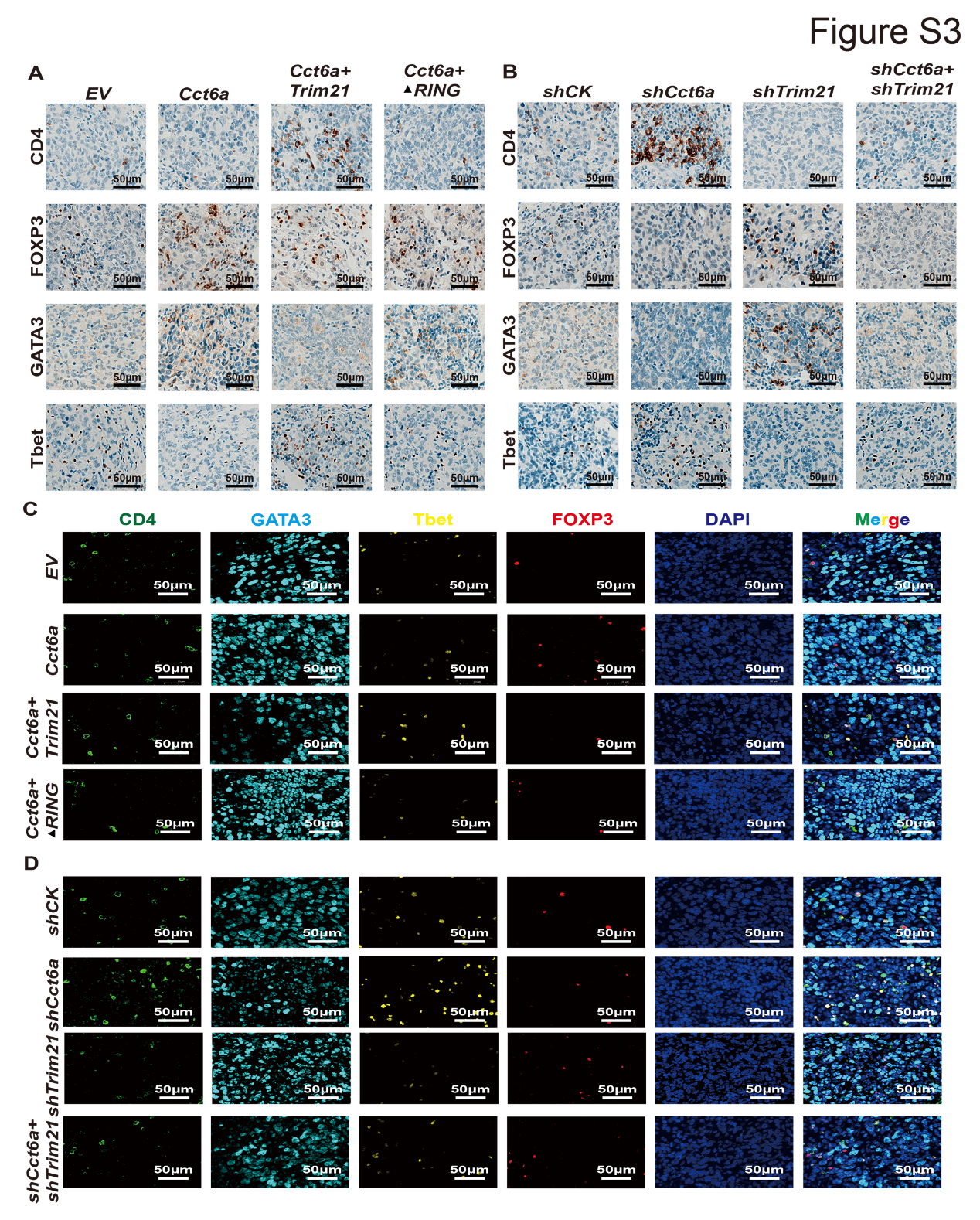
**

**Figure S3. TRIM21-mediated ubiquitination and degradation of CCT6A impacts the tumor immune microenvironment**

4T1 cells expressing *EV* (control), *Cct6a, Cct6a+Trim21*, or *Cct6a+Trim21-ΔRING* as indicated were injected into mice through the tail vein (n=5). Representative images of IHC(A) staining and Multiplex Immunofluorescence (C) for CD4, FOXP3, GATA3, and Tbet were stained in indicated lung metastasis tissues. 4T1 cells expressing *shCK* (control), *shCct6a, shTrim21*, or *shCct6a+shTrim21* as indicated were injected into mice through the tail vein (n=5). Representative images of IHC (B) staining and Multiplex Immunofluorescence (D) for CD4, FOXP3, GATA3, and Tbet were stained in indicated lung metastasis tissues.

**
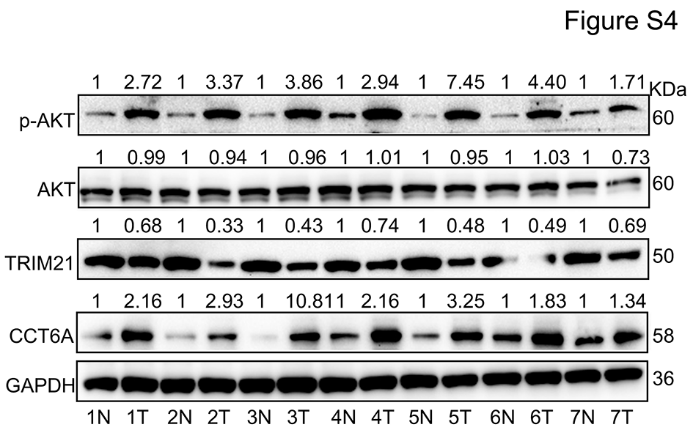
**

**Figure S4. The expression of TRIM21/CCT6A/AKT signal in TNBC tissues**

TRIM21, CCT6A and p-AKT/AKT expression was evaluated in TNBC tissues via western blot.

**
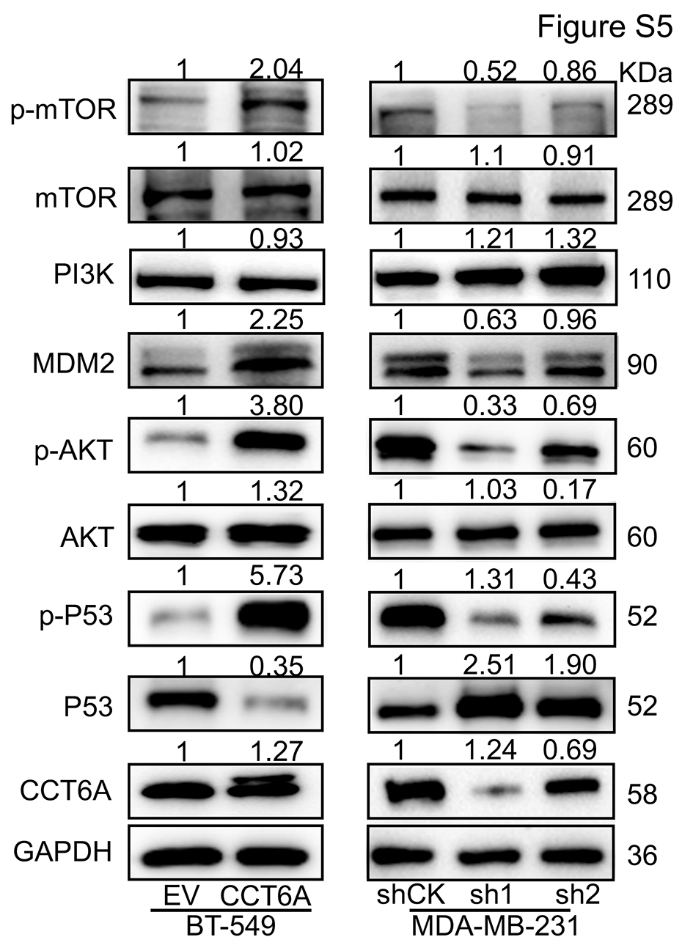
**

**Figure S5. CCT6A activates PI3K/AKT signaling pathway in TNBC cell lines**

p-AKT/AKT, p-mTOR/mTOR, MDM2 and p-P53/P53 expression was evaluated in BT-549 cells expressing CCT6A or EV control and MDA-MB-231 cells treated with shCK or shRNAs via western blot.

Supplementary Table 1. The target sequence of shRNA used in the study

| shRNA | Target sequence |
| --- | --- |
| shCCT6A-1 | CGTGTCATTAGAGTATGAGAA |
| shCCT6A-2 | CCAGAACATCTCTTCGTACTA |
| shTRIM21-1 | GAGTTGGCTGAGAAGTTGGAA |
| shTRIM21-2 | CTGGCATGGTCTCCTTCTACAA |
| *shCct6a*-1 | CGTGTCCTTAGAGTATGAGAA |
| *shCct6a*-2 | GACTCAAATCAAGGATGCAAT |
| *shTrim21*-1 | GGTTTGCAGAGATTCTGTTCA |
| *shTrim21*-2 | GGAAAGAGTTGGCCGAGAAGA |

Supplementary Table 2. Primers used in the study

| Genes | Sequence |
| --- | --- |
| CCT6A-Forward | ACACTCACTCAGATCAAAGATGC |
| CCT6A-Reverse | CCCTTTACACTGGGCTTATGTTT |
| E-Cadherin-Forward | CGAGAGCTACACGTTCACGG |
| E-Cadherin-Reverse | GGGTGTCGAGGGAAAAATAGG |
| Vimentin-Forward | GACGCCATCAACACCGAGTT |
| Vimentin-Reverse | CTTTGTCGTTGGTTAGCTGGT |
| N-Cadherin-Forward | GTTCCTGGAGCATGTACTTC |
| N-Cadherin-Reverse | CTTCCTCTTTGGGATTGTCC |
| snail-Forward | ACTGCAACAAGGAATACCTCAG |
| snail-Reverse | GCACTGGTACTTCTTGACATCTG |
| ZEB1-Forward | CAGCTTGATACCTGTGAATGGG |
| ZEB1-Reverse | TATCTGTGGTCGTGTGGGACT |
| TRIM21-Forward | TCAGCAGCACGCTTGACAAT |
| TRIM21-Reverse | GGCCACACTCGATGCTCAC |
| CDKL2- Forward | TCTCCCAGTCTGGCGTTGT |
| CDKL2-Reverse | ACCATCGGGTTGCCACATAAT |
| CDK18- Forward | TCTCCCAGTCTGGCGTTGT |
| CDK18-Reverse | ACCATCGGGTTGCCACATAAT |
| TP53- Forward | CAGCACATGACGGAGGTTGT |
| TP53-Reverse | TCATCCAAATACTCCACACGC |
| P21- Forward | TGTCCGTCAGAACCCATGC |
| P21-Reverse | AAAGTCGAAGTTCCATCGCTC |
| P16- Forward | GATCCAGGTGGGTAGAAGGTC |
| P16-Reverse | CCCCTGCAAACTTCGTCCT |
| CCND1- Forward | GCTGCGAAGTGGAAACCATC |
| CCND1-Reverse | CCTCCTTCTGCACACATTTGAA |
| BAX-Forward | CCCGAGAGGTCTTTTTCCGAG |
| BAX-Reverse | CCAGCCCATGATGGTTCTGAT |
| BCL2-Forward | GGTGGGGTCATGTGTGTGG |
| BCL2-Reverse | CGGTTCAGGTACTCAGTCATCC |
| GAPDH-Forward | GGAGCGAGATCCCTCCAAAAT |
| GAPDH-Reverse | GGCTGTTGTCATACTTCTCATGG |

CGGTTCAGGTACTCAGTCATCC

| \| GGTGGGGTCATGTGTGTGG \| \| --- \| |
| --- | --- |

Supplementary Table 3. Multivariate analysis for OS.

| Variable | OS | | |
| --- | --- | --- | --- |
|  | OR | 95%CI | p-value |
| TRIM21 | 0.017 | 0.001-0.512 | 0.019 |
